# Security-Aware AI for 6G LEO Satellite–Terrestrial Networks: Handover-Aware Temporal Graph Learning for Identity–Route Desynchronization Attacks

**DOI:** 10.3390/s26216846

**Published:** 2026-10-29

**Authors:** Mohammad Al-Omar, Loie Nimrawi, Wardeh Alrefai, Ala Mughaid

**Affiliations:** 1 Department of Cybersecurity, Faculty of Science and Information Technology, Irbid National University, Irbid 21110, Jordan; w.alrefai@inu.edu.jo; 2 Department of Computer Networks and Cybersecurity, Jadara University, Irbid 21110, Jordan; l.alnamrawi@jadara.edu.jo; 3 Computer Science Department, Gulf University for Science and Technology, Hawally 32093, Kuwait; 4 Department of Information Technology, Faculty of Prince Al-Hussein bin Abdullah II for Information Technology, The Hashemite University, Zarqa 13133, Jordan

**Keywords:** 6G satellite–terrestrial networks, Identity–Route Desynchronization Attack, Handover-Aware Temporal Graph Transformer, LEO satellite cybersecurity, temporal graph learning

## Abstract

Security-aware artificial intelligence is becoming essential for protecting future wireless communication networks, particularly 6G terrestrial–non-terrestrial networks (TN–NTNs), where rapid mobility and dynamic routing create new opportunities for cross-layer attacks. This study introduces a Handover-Aware Temporal Graph Transformer (HATGT) for AI-driven detection and forecasting of Identity–Route Desynchronization Attacks (IRDAs) in LEO satellite–terrestrial networks. A 6G-oriented TN–NTN simulation environment is constructed using ns-3.48 and 5G-LENA v5.1 with available NR/NTN modeling capabilities, where “6G-oriented” denotes prospective satellite–terrestrial integration characteristics rather than a finalized 6G air interface or operational deployment, incorporating heterogeneous UE mobility, terrestrial gNBs, LEO satellites, NTN gateways, handover dynamics, routing evolution, and session-state transitions. Five IRDA mechanisms are modeled, and 100,000 synchronized observations are generated from 60 independent simulation runs. HATGT represents wireless-network states as heterogeneous temporal graphs and integrates relation-aware graph encoding, explicit handover-aware gating, causal temporal attention, and supervised contrastive learning to capture security-relevant dependencies among mobility, session, anchor, and forwarding states. The shared representation jointly supports six-class attack attribution, binary IRDA detection, 5 s early-warning prediction, and time-to-attack estimation. Under a run-disjoint evaluation protocol involving independent stochastic realizations of a common network configuration, HATGT achieves 98.42% accuracy, 98.38% Macro-F1, 0.998 AUROC, and 0.992 AUPRC for multiclass detection. The early-warning head achieves 90.29% F1, 0.914 AUPRC, a 0.15% false-alarm rate, and an average warning lead time of 3.76 s. These simulation-based findings demonstrate the potential of handover-aware temporal graph learning to distinguish legitimate mobility-induced transients from malicious identity–route inconsistencies under the investigated TN–NTN conditions. The reported performance supports within-configuration stochastic generalization rather than verified cross-topology robustness or operational 6G deployment readiness. Further evaluation across diverse network configurations, prediction horizons, hardware-in-the-loop platforms, and operational TN–NTN testbeds is required to establish real-world applicability and end-to-end security-response feasibility.

## 1. Introduction

Sixth-generation (6G) wireless communication is expected to rely increasingly on integrated terrestrial–non-terrestrial network (TN–NTN) architectures in which Low Earth Orbit (LEO) satellite constellations complement terrestrial infrastructure to provide wide-area, low-latency, and resilient connectivity [1]. Such integration couples radio access, satellite backhaul, transport, core-network functions [2], and edge services across heterogeneous wireless domains [3]. Unlike conventional terrestrial networks, these environments experience continuously varying geometry, propagation conditions, elevation angles, Doppler effects, and link availability, causing access associations and forwarding states to evolve rapidly over time [4]. These characteristics make future satellite–terrestrial systems attractive application domains for artificial intelligence (AI), but they also create complex security conditions in which intelligent defense mechanisms must interpret rapidly changing wireless states without confusing legitimate dynamics with malicious behavior.

Mobility management represents one of the most security-sensitive processes in LEO-enabled wireless networks because satellite motion continuously changes serving and candidate access relationships [5]. Vertical handovers between terrestrial and satellite access depend on received signal strength, signal quality, elevation, mobility, and network conditions [6], while inter-beam and inter-satellite transitions are additionally influenced by coverage, propagation loss, network load, and topology dynamics [7]. Consequently, a handover is not an instantaneous association change but a temporal process involving preparation, execution, session migration, route modification, and subsequent state convergence. Temporary discrepancies among control, session, gateway, and forwarding states can therefore occur during legitimate mobility, creating an important challenge for security-aware AI: abnormal network behavior must be identified without interpreting every transient handover inconsistency as a cyberattack.

The dynamic and predictable structure of LEO communication also expands the wireless attack surface. Highly interconnected satellite constellations and predictable routing behavior can expose forwarding infrastructure to coordinated attacks, congestion, and denial-of-service conditions [8], while time-varying topology itself can be exploited to disrupt communication resources [9]. Secure-routing research further demonstrates that malicious behavior can influence forwarding decisions and requires continuous monitoring of routing and trust states [10]. A more subtle threat emerges when the wireless identity, mobility, or session state indicates one valid configuration while the active forwarding route remains stale, delayed, or maliciously redirected. Such identity–route desynchronization can remain difficult to recognize because individual radio, gateway, session, and routing observations may appear legitimate. Effective AI-based defense therefore requires joint reasoning across wireless mobility, handover progression, identity/session context, anchor association, and forwarding evolution rather than reliance on isolated traffic anomalies.

Graph-based AI provides a suitable foundation for this problem because satellite–terrestrial communication naturally forms a heterogeneous and continuously evolving relational structure. Graph neural networks can represent changing LEO topology and communication relationships among interacting network entities [11], while heterogeneous graph models preserve distinct entity and relation semantics that conventional feature-vector representations may obscure [12]. Dynamic graph Transformers additionally enable structural and temporal dependencies to be learned jointly, allowing anomalous behavior to be identified from abnormal evolution across consecutive network states [13]. For wireless cybersecurity, this capability is particularly important because a UE, satellite, gateway, or forwarding path may individually appear valid while their temporal relationship becomes inconsistent. Security-aware graph learning can therefore provide a stronger basis for distinguishing legitimate mobility evolution from coordinated cross-layer attacks.

AI-based wireless security should also move beyond reactive binary intrusion detection toward richer threat characterization and anticipatory defense. Multi-task learning can exploit shared security representations while preserving task-specific decisions [14], whereas predictive cybersecurity models demonstrate that future security conditions can be estimated before an attack becomes fully observable [15]. Temporal graph forecasting further shows that historical network interactions can support prediction of future malicious events and affected entities [16]. Combining attack attribution, present-state detection, early-warning prediction, and time-to-event estimation is therefore particularly valuable in highly mobile wireless environments, where defensive mechanisms must determine not only whether suspicious behavior is occurring, but also how it is evolving and how soon a significant security state may emerge.

The main contributions of this study are summarized as follows:

- A 6G TN–NTN security simulation is developed using ns-3.48 and 5G-LENA v5.1, covering heterogeneous mobility, terrestrial/LEO access, four handover types, five IRDA mechanisms, and 100,000 observations from 60 runs.
- A Handover-Aware Temporal Graph Transformer (HATGT) is developed using relation-aware graph encoding, handover-aware gating, and causal temporal attention to capture spatial–temporal identity and routing dependencies.
- A unified AI security framework jointly performs six-class IRDA attribution, binary detection, 5 s early-warning prediction, and time-to-IRDA estimation through contrastive and multi-task learning.

## 2. Literature Review

Azar et al. [17] explored machine and deep learning approaches to intrusion detection for satellite networks to enhance detection of attacks while simultaneously reducing the dimensionality of the features and computational complexity. They discussed the challenges of taking traditional IDS techniques to the satellite environment. The authors compared the performance of the classifiers Random Forest, ANN, LSTM, and GRU with a model that was based on the Random Forest classifier and Sequential Forward Selection. The authors used Random Forest-based Sequential Forward Selection with the Random Forest, ANN, LSTM and GRU classifiers and tested the models on STIN and UNSW-NB15 datasets. On STIN, SFS-RF achieved 90.50% accuracy, 91.19% precision, 90.41% recall, and 90.00% F1-score using only 10 features, while RF-SFS-GRU improved accuracy from 79.00% to 87.00%. The approach does not model the temporal consistency of mobility, handover, session, gateway, and forwarding-route states needed for IRDA detection; however, it carries out traffic-level classification.

Driouch et al. [18] introduced CANSat-IDS, a distributed intrusion-detection system to safeguard satellite Controller Area Network communications in a resource-limited satellite environment. They aimed to decrease the amount of work the spacecraft needed to do while keeping attacks detected effectively. The architecture involves distributing the lightweight anomaly detection to the satellite and more computationally demanding CAN-frame analysis to the ground station, with machine learning classifiers being used to recognize attacks. The results obtained from the experimental evaluation under varying attack conditions showed F1-scores between 91.12% and 99.86%. However, the framework is limited to attacks on the onboard CAN and does not cover TN–NTN UE mobility, handover phases, gateway associations and synchronization of identity/session and forwarding state.

However, for satellite network intrusion detection, Shi et al. [19] introduced the concept of BLSAE-SNIDS, which aims to tackle the challenges of limited access to labeled data, computational constraints, and maintaining the sequential nature of network traffic. They use a bidirectional LSTM and a stacked sparse autoencoder to first learn compressed representations of the time series, before attacking the classification of the attacks. The framework was experimentally tested on two datasets: STIN and UNSW-NB15. The accuracy, precision, recall, and F1-score of BLSAE-SNIDS-DNN on STIN had been 99.99. Although it performs well, the method focuses primarily on learning the sequential traffic pattern and does not explicitly describe UEs, satellites, gateways, anchors, routes and handover states as heterogeneous relationships, which is a drawback to identify identity–route desynchronization.

He et al. [20] introduced STINIDF for enhancing intrusion detection in the case of heterogeneous satellite–terrestrial traffic distributions. They looked at non-IID data at distributed STIN nodes, where the federated IDS performance can be impaired. It jointly trained a conditional diffusion model through federated learning and differential privacy, then integrated synthetic data generated by the global model with local traffic to classify intrusions. The experiments performed with an OMNeT++/INET STIN environment achieved 96.63% accuracy, 96.71% precision, 96.54% recall, and 96.66% F1-score, whereas the privacy-preserving configuration was able to achieve 96.14% accuracy. The approach does not deal with temporal inconsistencies between handover, session and anchor and routing states but rather with traffic attacks and distribution heterogeneity.

To detect intrusions quickly in Space–Air–Ground Integrated Networks (SAGINs) with minimal computational workload at the satellite and lower intrusion delay, Xie et al. [21] designed the SAGIN ID system. They studied satellite-limited processing and the requirement for speedy security reply in dynamic SAGIN environment. It is an integration of smart-contract based matching on board, an early-stop optimized semi-supervised voting algorithm, and a distributed ground processing based on Flink. A simulated SAGIN environment was used to generate six categories of DoS related attacks and tested in four experiments. The intrusion detection accuracy of SAGIN-ID was about 99% and it decreased the delay time and reduced the amount of satellites resources used. However, it can be used to detect traffic-oriented attacks, yet it fails to model mobility-dependent synchronization between identity/session, handover, gateway, and forwarding-route states.

Liu and Guo [22] proposed DIGNN-A, a dynamic graph neural network for real-time network intrusion detection. Their study addressed the inability of conventional IDS models to represent evolving communication topology and dependencies among interacting network entities. The method converts traffic into dynamic graph representations, applies graph convolution and multi-head attention, and uses line-graph transformation for fine-grained attack classification. Experiments on UNSW-NB15 and NF-ToN-IoT-v2 achieved 99.30% and 99.96% accuracy, respectively, with a false-alarm rate of 0.0003 on NF-ToN-IoT-v2. Nevertheless, DIGNN-A uses generic communication graphs and does not explicitly model TN–NTN handover phases, serving/candidate access relationships, control/data anchors, or identity–route synchronization.

Dewa et al. [23] investigated graph-based handover management in LEO satellite networks to reduce unnecessary mobility transitions and improve user assignment. Their research addressed frequent satellite movement, dense user populations, and the complexity of optimal satellite–terminal association. A distributed message-passing algorithm was developed to exchange local information between satellites and users and determine near-optimal handover assignments. MATLAB R2026b experiments with 500 users achieved 2.79 handovers per user, representing an 18.89% improvement, while providing approximately $0.48\times 10^{8}$ bps/user and a satisfaction value of 0.79. However, the framework optimizes legitimate handover decisions rather than identifying malicious post-handover inconsistencies between session context and forwarding routes.

Naz et al. [24] proposed GNN-TASR for trust-aware secure routing in dynamic LEO satellite networks. Their objective was to overcome rapidly changing topology and malicious or selfish satellite behavior that reduce the reliability of conventional routing approaches. The framework uses a Message-Passing Neural Network to learn trust from link availability, honesty, and selfishness, followed by trust-constrained Dijkstra routing. Simulations achieved 98% malicious-node detection accuracy for OneWeb and approximately 93% for Telesat, together with a 94% packet-delivery ratio, 30% lower delay, and 22% higher throughput. However, the method identifies malicious nodes and trusted routes rather than temporal inconsistencies between otherwise legitimate UE identity/session and forwarding states.

Uddin et al. [25] proposed HSP-SR, a hierarchical privacy-preserving intrusion-detection framework for SAGIN-enabled IIoT environments. Their study addressed heterogeneous communication links, mobility, constrained resources, and cross-tier attack propagation. The framework combines ground-level autoencoder detection, air-tier attention-based trust aggregation, space-tier time-varying GNN reasoning, and Deep Q-Network-based mitigation. Experiments using CSE-CIC-IDS2018 under six attack scenarios achieved 99.42% detection accuracy, a 0.72% false-positive rate, 22.1% lower energy consumption, and detection latency below 130 ms. However, it addresses general cross-tier intrusion propagation rather than handover-dependent synchronization between UE identity/session, gateway, and forwarding-route states.

Yao and Koirala [26] developed DTGNN-FIDS for fault-tolerant intrusion detection in dynamic IIoT–5G environments. Their study addressed evolving topology, adversarial attacks, and simultaneous node or edge failures. The framework represents traffic as temporal graphs and combines fault-modulated graph attention, contrastive learning, adversarial regularization, fault-consistency optimization, and edge–cloud computation offloading. Experiments on Edge-IIoTset, ToN-IoT, and IoT-23 maintained Macro-F1 scores of at least 92.4% for fault rates up to 20%, achieved 91.3% adversarial robustness, 41.8 ms inference latency, and reduced bandwidth overhead by 68%. However, it does not model LEO/TN–NTN handover semantics, identity–route synchronization, or jointly perform current IRDA classification, future-onset prediction, and time-to-IRDA estimation.

Table 1 shows that the existing literature addresses important but largely separate aspects of satellite and dynamic-network security, including traffic-level intrusion detection, onboard CAN protection, federated and privacy-preserving learning, generic dynamic graph analysis, handover optimization, trust-aware routing, and cross-tier intrusion mitigation. However, the reviewed approaches do not jointly represent the temporal consistency among UE mobility, handover progression, session state, gateway or anchor association, and forwarding-route evolution in an integrated LEO TN–NTN setting. This gap is particularly important for Identity–Route Desynchronization Attacks, where individual network states may remain locally plausible while their cross-layer temporal relationships become inconsistent. The proposed HATGT framework addresses this gap through heterogeneous relation-aware graph modeling, explicit handover-aware state encoding, causal temporal learning, and coordinated multiclass detection, binary detection, 5 s early-warning prediction, and time-to-IRDA estimation.

## 3. Methodology

**Figure 1:**
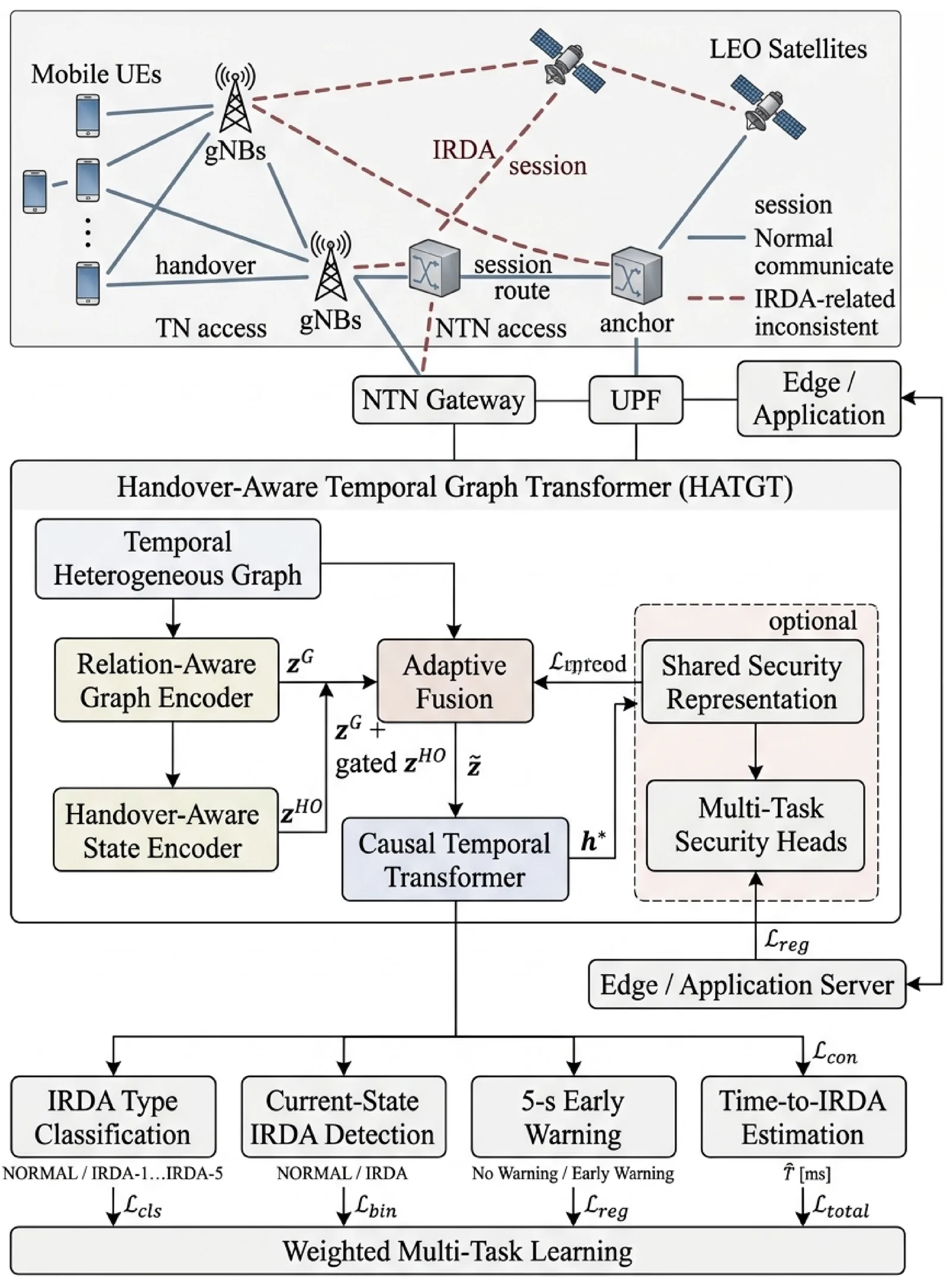
Proposed methodology.

The proposed methodology integrates 6G-oriented terrestrial–non-terrestrial networking, Identity–Route Desynchronization Attack (IRDA) modeling, and handover-aware temporal graph learning within a unified security-analysis framework. As illustrated in Figure 1, mobile UEs communicate through terrestrial gNBs and LEO satellites while mobility, handover, session, routing, and anchor states evolve across TN and NTN access domains. Legitimate communication is represented through normal serving, handover, gateway, UPF, and edge-service paths, whereas IRDA is characterized by temporally inconsistent session, route, and anchor relationships rather than by an inherently malicious physical node. The resulting cross-layer state is mapped into the proposed Handover-Aware Temporal Graph Transformer (HATGT), whose core combines a temporal heterogeneous graph representation with a relation-aware graph encoder and a dedicated handover-aware state encoder. Their outputs, $z^{G}$ and $z^{HO}$, are integrated through adaptive gated fusion before being processed by a causal temporal Transformer to obtain the shared security representation $h^{*}$. This representation supports four coordinated security objectives: six-class IRDA attribution, binary current-state detection, 5 s early-warning prediction, and time-to-IRDA estimation. The corresponding losses are jointly optimized through weighted multi-task learning, while supervised contrastive learning further strengthens separation between NORMAL behavior and distinct IRDA mechanisms.

### 3.1. 6G-Oriented LEO Satellite–Terrestrial Network Simulation and IRDA Scenario Generation

The experimental environment was constructed using Ubuntu Linux, ns-3.48, and 5G-LENA v5.1 to establish a 6G-oriented integrated terrestrial–non-terrestrial network (TN–NTN) simulation based on available 3GPP NR and NTN modeling capabilities. The term 6G-oriented denotes the anticipated integration of terrestrial radio access, LEO satellite connectivity, mobility-aware routing, and edge-assisted services rather than implementation of a finalized 6G air-interface standard. The topology comprises 60 mobile user equipments (UEs), three terrestrial gNBs, six LEO satellites, two NTN gateways, one User Plane Function (UPF), and two edge/application servers. The terrestrial segment operates at approximately 3.5 GHz with 20 MHz bandwidth, numerology μ=1, TDD operation, and a nominal gNB transmission power of approximately 43 dBm, whereas the NTN segment adopts a LEO altitude of approximately 550 km, a representative carrier frequency of approximately 700 MHz, and approximately 5 MHz bandwidth. The satellite component incorporates time-varying UE–satellite distance, elevation angle, propagation delay, Doppler variation, evolving coverage, and satellite–gateway association. Heterogeneous mobility was introduced by assigning approximately 12% of the UEs to static behavior, 48% to pedestrian mobility at 0.8–2.0 m/s, and 40% to vehicular mobility at 7–27 m/s. Legitimate mobility transitions were generated across TN→TN, TN→NTN, NTN→TN, and NTN→NTN access combinations using serving- and candidate-node RSRP, RSRQ, and SINR measurements, together with an approximately 3 dB handover hysteresis and an approximately 80 ms Time-to-Trigger. Each transition evolves through STABLE, PREPARATION, EXECUTION, and RECOVERY phases, while handover timing, serving and candidate access nodes, route evolution, gateway association, and session-state changes are continuously recorded. TELEMETRY, CONTROL, VIDEO, and BACKGROUND traffic profiles are generated over TCP and UDP to introduce realistic variations in throughput, RTT, jitter, queueing delay, packet loss, retransmission rate, and concurrent flow activity.

This simulation campaign consists of 60 independent stochastic realisations with a common reference TN–NTN configuration. In all the scenarios, the numbers of UEs, terrestrial gNBs, LEO satellites, NTN gateways and edge servers are also constant. Other factors like the underlying topology-generation framework, radio configurations, mobility-category proportions, traffic profiles, handover rules, and IRDA attack-generation mechanisms are also consistently kept. The seeds are used to introduce variance of actual network dynamics such as UE mobility, access associations, handover sequence, traffic variation, routing evolution and the timing of the attack events, into the simulated network within the specified simulation parameters. As a result, the individual runs are stochastic realizations of the same experimental configuration, not different network topologies, or significantly different deployment scenarios. The evaluation protocol used to evaluate the model is the  run-disjoint protocol that divides the 42 simulation runs into a training set of 33 runs, a validation set of 9 runs, and a testing set of 9 runs, such that observations from the same model realization are not presented in more than one set. This separation helps to evaluate the process of generalization to unseen stochastic realizations in the investigated network configuration while minimizing the risk of leakage of run-level information. It does not establish the generalization across various sizes of satellite constellations, densities of UE, deployment of terrestrial access, placement of gateway, mobility distribution, traffic-generating configuration and/or attack-generating configuration. The within-configuration stochastic generalization is thus described, not the cross-topology or the cross-configuration robustness.

**Figure 2:**
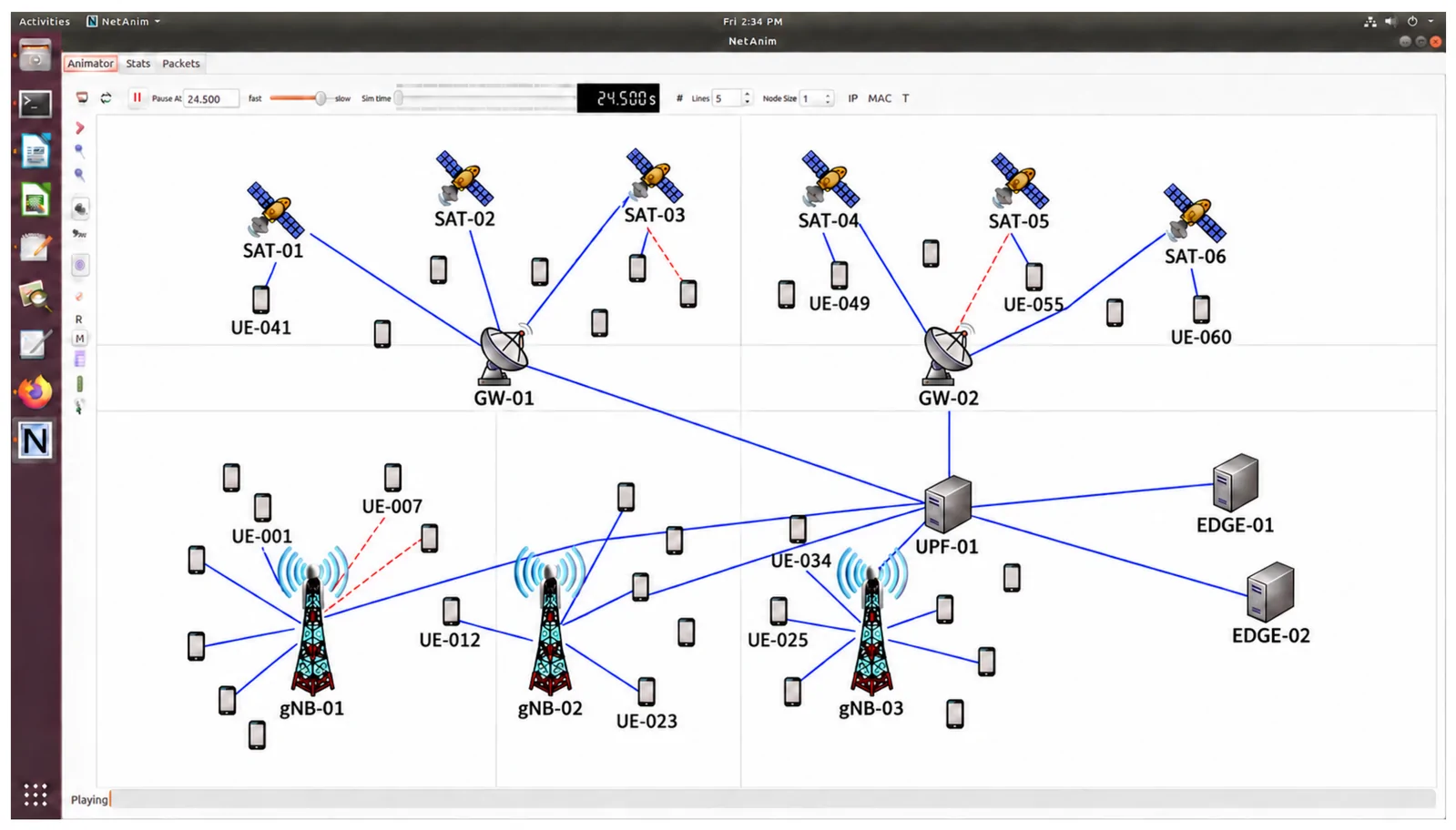
NetAnim visualization of the ns-3.48/5G-LENA 6G-oriented TN–NTN simulation environment, including mobile UEs, terrestrial gNBs, LEO satellites, NTN gateways, the UPF, and edge/application servers employed for mobility, handover, traffic, and IRDA scenario generation.

Figure 2 illustrates the simulated TN–NTN topology and the interaction of its terrestrial, satellite, core-network, and edge-service components. Mobile UEs establish access through the terrestrial gNBs or LEO satellites according to their radio and mobility conditions, while NTN traffic is relayed through the corresponding gateways and both terrestrial and non-terrestrial forwarding paths ultimately reach the UPF and edge/application servers. Within this dynamic environment, the Identity–Route Desynchronization Attack (IRDA) model disrupts the temporal consistency between UE identity/session/mobility context and the forwarding state associated with its traffic. Five IRDA mechanisms are considered: stale-context replay, gateway-binding manipulation, handover-state injection, delayed route synchronization, and coordinated low-rate desynchronization. Legitimate executions are also configured to include short route-convergence delays, temporary control/data-path differences, congestion, aborted handover preparation, satellite-coverage transitions, and session recovery, ensuring that ordinary mobility-induced fluctuations overlap with potentially malicious behavior.

### 3.2. TN–NTN IRDA Dataset Construction, Feature Characterization, and Temporal Representation

The TN–NTN IRDA dataset employed in this study was generated specifically from network observations produced by the ns-3.48 simulation environment and the 5G-LENA v5.1 NR framework. The simulation design and interpretation of terrestrial–non-terrestrial connectivity follow the NR-NTN principles established by 3GPP. Accordingly, the dataset is not derived from an existing intrusion-detection benchmark; instead, it was constructed from synchronized observations of simulated radio, mobility, handover, topology, routing, traffic, and session states so that Identity–Route Desynchronization Attack behavior could be analyzed within its intended mobility-dependent TN–NTN context. Although the complete simulated topology contains 60 active UEs, 50 UEs, identified as UE-001–UE-050, were designated as monitored learning entities from which labeled UE-state observations were retained. The remaining 10 UEs continued to participate in the simulated environment as background mobility and traffic entities and therefore contributed to network load, interference, congestion, association changes, and handover dynamics without being retained as labeled learning instances. The observations originate from 60 independent simulation runs identified by run_id=1, …, 60, with reproducible random seeds ranging from 900001 to 900060. For each monitored UE, 100 synchronized observation instants were defined between 0.0 and 49.5 s at a nominal resolution of 0.5 s. Consequently, the 60 runs, 50 monitored UEs, and 100 observation instants provide an upper candidate pool of 60×50×100=300,000 UE-state records before validation, eligibility filtering, and class-aware sampling.

The same number of terrestrial gNBs, LEO satellites, NTN gateways, UEs, and edge servers, as well as the same radio parameters, mobility-category distribution, traffic generation framework, handover parameters, and five IRDA mechanisms were used for all 60 simulation runs. These provided conditions are further augmented with variations in the actual mobility trajectories, access associations, handover sequences, traffic conditions, routing evolution and attack-event timing generated by the introduction of independent simulation seeds. The collection of data was therefore not a significant difference in network topology or deployment, but rather, it was done as independent stochastic experiments of the same experimental configuration. This distinction is crucial in terms of generalizing the ability of the proposed HATGT framework as it does not guarantee robustness to unseen network configurations on its own, because runs of a simulation may be independent but the network configurations may not be.

The processing steps for constructions of the datasets were fixed, including timestamp synchronization, security-target construction, structural validation, feature extraction, categorical-state organization, eligibility filtering, class-aware sampling and chronological ordering. Out-of-sync candidate observations for UE, access-node, routing, anchor, and session context were filtered out prior to the construction of the learning dataset. These simulated schedules and state transition, along with the eligible observation pool, were then used to create security labels and then class-aware sampling without replacement was used on the labels and representation, while not being lost across the independent simulation runs. This balancing of the six classes results in a deliberately attack-enriched binary benchmark: When the five IRDA classes are aggregated, there are 83,330 malicious observations (83.33%) and 16,670 observations of NORMAL (16.67%). These proportions are based on the experimental sampling and not representative of an estimated IRDA incidence rate. Specifically, the binary distribution was balanced post-construction of the targets and post-construction of the candidate records, but prior to forming the temporal window and partitioning the final learning records. The resulting CSV dataset has 50 columns and a single row per monitored UE per observed time instant kept. The inspection of the datasets revealed that all 50 columns are present, no column contains missing values, there are no duplicate observation tuples in each dataset and no observation tuples in any dataset contain duplicated run_id–timestamp_s–ue_id observation tuples.

The 0.0–49.5 s range of timestamp_s is the local interval that is used to index the retained dataset, but it does not limit the upper bound of the state-history variables. Temporal attributes include total durations since the last handover, route age and session age, which are the total durations since the network state involved is last established during the commencement of the retained observation interval. These counters thus maintain the history of the existing network, and can reach more than 49.5 s even if each preserved record is within the 0.0–49.5 s local observation window. Examples of this include the elapsed duration of states (last-HO time), route age, and session age which reach 79.88 s, 153,472 ms, and 469.55 s, respectively, as they are computed from elapsed or accumulated state duration instead of from the absolute dataset timestamp. When considering future event variables, these are also not interpreted as alternate values of the timestamp_s rather as future events and event sequence from the corresponding UE attack schedule. This separation is used to keep the routing, session and mobility histories real while keeping the interval synchronized for model observation and temporal learning in a compact interval.

The 50 dataset columns consist of 46 describing the experimental state of the network and the network itself, and four that are learning targets. The non-target attributes are grouped together as metadata, access/topology, radio and NTN, mobility, handover, routing, traffic/QoS, and session-state groups. Instead of encoding an explicit binary feature that can signal identity directly—route inconsistency—the dataset keeps the primitive network states from which these inconsistencies need to be learned. The variables is_desynchronized, is_anchor_mismatch, attack_score, expected_gateway and ground_truth_attack_state are intentionally not included in the predictive feature space. The metadata identifiers, such as run_id, seed and ue_id are kept for the purposes of reproducibility, UE-level trajectory reconstruction, and leakage-safe partitioning but do not provide direct attack signatures. In the case of temporal learning, the observations are collected into groups based on simulation run and UE and then sorted by timestamp_s.

For UE *u*, a history ending at time *t* is represented as

$$S_{u,t}=\left\{x_{u,t-L+1},\ldots ,x_{u,t}\right\},$$

where *L* denotes the temporal-window length selected during model configuration. No temporal sequence is permitted to cross either a UE boundary or an independent simulation-run boundary. This representation preserves the evolution of serving and candidate links, handover state, route state, anchor relationships, session progression, and QoS conditions rather than treating each retained observation as an independent tabular instance. Each of the  60 independent simulation runs is grouped into mutually exclusive training, validation and testing subsets of length  42,  9 and  9, respectively. Thus, observations from the same stochastic simulation run are not permitted to be in different learning partitions. Numerical normalization and categorical transformations are fit solely on the training partition, so as to not leak any information and maintain experimental independence at the pre-processing level. The resulting partitions enable assessment of generalization for unseen stochastic realizations in the same one-to-one topology–one-to-n topology configuration, but not in cross-topology or cross-configuration generalization.

The cross-layer 6G-oriented TN–NTN experimental environment is summarized in Table 2 with the network population being distinguished from the retained learning population to emphasize the difference. The complete topology includes 60 UEs and 50 monitored UEs provide the labeled observations for the learning data set. These entities are monitored over 60 independent runs, and 100 synchronized observation instants, where structural validation, eligibility filtering and target construction, and class-aware sampling lead to 100,000 monitored UE-state records. The terrestrial segment is comprised of three gNBs operating close to 3.5 GHz with 20 MHz bandwidth, numerology μ=1 (smaller numerology), TDD operation, and approximately 43 dBm transmit power, while the NTN segment includes six LEO satellites at approximately 550 km altitude, 700 MHz carrier frequency, 5 MHz bandwidth, two gateways, propagation delays of 0.318–6.251 ms, Doppler shifts up to ±19 kHz, elevation angles of 0–85° and serving distances up to 1700 km. Mobility behavior encompasses static, pedestrian and vehicular users, with pedestrian speed ranging from 0.8–2.0 m/s and vehicular speed ranging from 7–27 m/s. These handover dynamics are controlled by about 3 dB hysteresis, nominal TT time of 80 ms, dataset TT times of 80/100/128/160 ms, A3 margin of −21.15–2.97 dB, and STABLE, PREPARATION, EXECUTION and RECOVERY phases during TN–TN, TN–NTN, NTN–TN and NTN–NTN transitions. The table also separates the local 0–49.5 s dataset timestamp from other temporal-state indicators like time since last handover, route age and session age that are not limited by the local observation interval, but are capable of holding the history of previous state. The UE/GNB/SAT/GW/UPF/EDGE graphs are mapped into heterogeneous graphs and processed by relation-aware encoding, handover-aware gating, causal multi-head attention, supervised contrastive learning, and four heads for 6-class attribution, binary detection, 5 s forecasting, and time-to-IRDA regression.

The 5 S early-warning objective has an imbalance ratio of  57.21:1, with 1718 positive precursor observations and 98,282 negative observations. Positive observations suggest that an IRDA onset would happen within the next 5 s, and class-weighted forecasting loss $L_{pred}$ alleviates this significant imbalance. Importantly, these 1718 positive observations are not attack events, but rather time stamp-level precursors to attacks; that is, multiple consecutive observations could occur from the same attack onset in the near future. The time-to-IRDA regression target is defined on interval of time $T_{u,t}\in \left[0,59,996\right]$ ms and is highly skewed with 83,330 zero values (83.33%) and 16,670 strictly positive values (16.67%). Zero valued targets are observations when the remaining pre-onset interval was not positive; positive targets indicate the number of milliseconds to a future IRDA activation (500 to 59,996 ms). This difference is important because if the majority of the targets are zero, it can have a significant impact on the aggregate regression-error measures. The loss function is Smooth-L1 loss, and the target transformation is a log(1 + T) transform which ensures that the target is not negative. The loss function is Smooth-L1 loss and the target transformation is a log(1 + T) transform so that the target is not negative. HATGT learns the attack attribution, current-onset IRDA detection, future-onset prediction, and time-to-IRDA estimation tasks together within a single multi-task optimization framework, using the shared representation of time.

A summary of the four coordinated security goals of HATGT is presented in Table 3 to illustrate the difference between the proposed multi-task architecture and the typical single output intrusion classifiers. For current-state binary detection, target_binary_irda combines the five IRDA mechanisms into 83,330 observations of malware (83.33%) and 16,670 observations of NORMAL (16.67%). The five IRDA categories consist of about the same number of observations, not the naturally occurring attack prevalence in operational TN–NTN situations, but a deliberately constructed benchmark for controlled attack-attribution analysis. These reported binary F1 scores, which range from  98–99%, are therefore not to be interpreted as the operational detection effectiveness, but rather as a measure of performance under the attack-enriched benchmark. Assuming the prevalence of IRDA to be 0.1%, and the conditional detection rate and false-positive rate to be constant, the expected precision is 5.22%, which means a false-discovery proportion of 94.78%. The prevalence adjusted estimates show that the positive predictive value and alert reliability varies considerably with the attack frequency and this variable is important under realistic operating conditions when reporting the precision, the false positive rate, the negative predictive value and the false alert rate (per 10,000 observations).

The regression target for the time-to-IRDA has a highly skewed distribution, with 83,330 zero-valued observations (83.33%) and 16,670 positive time-to-event observations (16.67%). These two target categories are clearly differentiated in regression, affect overall error measurements, and can be significantly affected by analysis, as their relative frequencies can significantly change. Zero-target samples construct a regression to represent the observations for which it was constructed. IRDA does not provide any positive remaining time to target. The positive-target samples have a measurable amount of observations, whereas the onset of the samples does not. The time interval between the corresponding IRDA events is considerable. The regression-learning procedure therefore needs to be able to differentiate the difference between predictions made by zero-target classifiers and related to positive targets of remaining time. In particular, the overall MAE of 381 ms is calculated for the entire population being evaluated and should not be taken as the prediction error related to only using positive time-to-event observations. Consequently, regression evaluation is additionally stratified by target category to distinguish zero-target classifiers. The temporal estimation accuracy vs prediction behavior is significant. To obtain these results, observations were performed with positive time-to-IRDA values.

The prevalence sensitivity is interpreted as a principal binary-detection finding. Let π=P(Y=1) denote the IRDA prevalence, TPR the true-positive rate, and FPR the false-positive rate at a fixed decision threshold. If these conditional error rates remain constant under a change in prevalence, the expected precision is

$$\mathrm{PPV}\left(\pi \right)=\frac{\pi \;\mathrm{TPR}}{\pi \;\mathrm{TPR}+(1-\pi )\;\mathrm{FPR}}.$$

Therefore, the attack base rate is also important for precision even if sensitivity and specificity do not change. The malicious prevalence of the primary benchmark (83.33%) is used to interpret the reported  98–99% binary F1-score for the sample distribution constructed in that work alone. The error rate described by the existing base-rate sensitivity analysis is instead projected at 5.22% for a prevalence of 0.1%, or 94.78% for the fixed-error-rate assumption. This is not an actual precision data, nor is it an actual IRDA incidence (but a low base-rate illustration) from an operational TN–NTN deployment. If there is no change in the observation unit, the threshold, and the definition of the FPR, then the number of expected false positives per 10,000 observations is 10,000(1−π)FPR. Observation-level false-positive counts should be differentiated from temporally correlated UE-state observations, which may result in repeated alerts, as well as from false alarms per unit time. In a real-world context, naturally representative base rates are needed, the thresholds need to be tuned with a different set of data, the probability needs to be calibrated, and the false alarm-workload and the alert-workload evaluation needs to be carried out during deployment. The operational F1 cannot be determined just from the benchmark F1 without providing the prevalence and conditional error rates.

The early-warning task constructs labels with respect to separately occurring IRDA onset events, instead of considering all malicious timestamps as individual events. Assume *e* is an IRDA episode for UE *u* in simulation run *r* and $t_{e}^{\mathrm{on}}$ is the first time that the attack mechanism for *e* is active, only if it includes evidence of an onset before *t* and one or more evidence of onset after *t*;

$$0<t_{e}^{\mathrm{on}}-t\leq 5\;s,$$

where both *t* and $t_{e}^{\mathrm{on}}$ are limited to the same run_id and ue_id trajectory. Samples after the activation of the attack are thus not taken as positive precursor samples for that attack. Due to the 0.5 s sampling interval, if a forthcoming IRDA event has a warning period of 5 s, then this event can give rise to multiple overlapping positive precursor timestamps in the warning period. Observations are saved in sequence learning as the underlying state changes over time but are aggregated together during the evaluation of the underlying attack.

To have an active IRDA period, onset is only identified when the period changes from non-IRDA to IRDA, and subsequent timestamps in the same continuous malware period are not new onsets. If the attack ends and a new independent IRDA episode starts, the new transition is considered a new event. If the manipulations in the coordinated low-rate desynchronization can take place in different stages of the network, then $t_{e}^{\mathrm{on}}$ is the first time the condition of the simulator is coordinated IRDA is activated. This definition distinguishes gradual precursor evolution from the eventual activation point, and ensures that each (low rate) manipulation timestamp does not count as an independent attack event.

Evaluation is carried out at the event level, which is done independently from the classification performed at the timestamp level. Consecutive positive predictions between same UE for same upcoming IRDA onset are merged into 1 warning episode. A successful warning of an attack event is deemed to have been made if at least one prediction is above the decision threshold and within the 5 s pre-onset window. Event-level recall is defined as the proportion of true attack-onset events receiving at least one valid pre-onset warning and event-level precision is defined as the proportion of warning episodes generated that correspond to a true IRDA onset within the next 5 s. The warning lead time is the time between the first valid warning of a detected event and the occurrence of the warning, and 

$$\Delta t_{e}=t_{e}^{\mathrm{on}}-t_{\mathrm{alert},e}^{\mathrm{first}}.$$

If multiple thresholds are crossed in the same warning interval, this is not considered a duplicate detected event, but rather a redundant warning interval is reported as duplicate alerts per detected event. Thus, the early-warning evaluation reports event-level metrics of precision, recall, F1, AUROC, AUPRC, and false alarms as well as the timestamp-level metrics of precision, recall, F1, AUROC, AUPRC, and false alarms.

The comparison methods were chosen for their progressive nature and their methodological diversity, covering conventional machine learning methods, sequential deep learning methods, graph learning methods, and temporal graph learning methods. As solid tree based baselines, Random Forest and XGBoost were chosen to evaluate the ability of static feature interactions alone to accurately classify IRDA states, with XGBoost being a popular gradient-boosting framework for high-dimensional structured data [27]. Sequential modeling was evaluated by comparing BiLSTM and GRU to determine how much sequential information can be gleaned from a series of observations across the network, without being explicitly aware of the underlying communication topology. GAT is integrated to examine the advantage of graph-based reasoning by aggregating with attention to neighbors, while Temporal GAT does so with both the graph relationships and the temporal dynamics taken into account. To separate the effects of temporal self-attention from an explicit heterogeneous graph structure, a Vanilla Transformer was also added, following the attention-based sequence modeling paradigm of Vaswani et al. [28]. Finally, the closest architectures to HATGT are the Temporal Graph Transformer, which similarly supports both graph based relational reasoning and temporal modeling, but without the proposed explicit encoding of the state for the handover and the handover aware fusion operation. The selected baselines are therefore not meant to suggest that all of the individual methods are the state of the art, but rather to provide an ideal mix of representative and competitive approaches from the main modeling families that are relevant to IRDA detection and forecasting, and allow for a systematic evaluation of the contributions of static, sequential, graph-based, temporal and handover-aware reasoning.

The temporal representation is constructed only after feature and target separation so that future-label information cannot enter the observation history. Let $x_{u,t}\in R^{d}$ denote the leakage-safe feature representation associated with UE *u* at time *t*. A temporal history of length *L* is defined as

$$S_{u,t}=\left[x_{u,t-L+1},x_{u,t-L+2},\ldots ,x_{u,t}\right],$$

with all observations restricted to the same run_id and ue_id. No temporal window is permitted to cross either a UE boundary or a simulation-run boundary. This constraint is essential because the end of one independent simulation realization and the beginning of another do not represent a physically continuous network trajectory. Categorical variables are transformed into model-compatible representations after dataset partitioning, while continuous variables are normalized using statistics estimated exclusively from the training partition. The resulting temporal samples preserve the progression from serving-link degradation and candidate-link improvement through handover preparation and execution to route/session stabilization, allowing short legitimate transient inconsistencies to be distinguished from persistent or malicious state divergence. This temporal organization also provides the observation histories required by the heterogeneous graph snapshots introduced in Section 3.3.

To preserve experimental independence, the final learning subsets are created according to simulation run rather than through random row-level sampling. If $R_{\mathrm{tr}}$, $R_{\mathrm{val}}$, and $R_{\mathrm{te}}$ denote the sets of simulation runs assigned to training, validation, and testing, respectively, the partitioning requirement is

$$R_{\mathrm{tr}}\cap R_{\mathrm{val}}=R_{\mathrm{tr}}\cap R_{\mathrm{te}}=R_{\mathrm{val}}\cap R_{\mathrm{te}}=⌀.$$

The corresponding training, validation, and testing partitions contain 42, nine, and nine independent simulation runs, respectively. This run-disjoint design prevents highly correlated observations originating from the same mobility trajectory, traffic realization, attack schedule, and route evolution from being distributed across multiple subsets. It provides a more stringent assessment of generalization to unseen stochastic realizations than conventional random row-level partitioning, since the test observations originate from simulation realizations that are completely absent during model fitting. Because all temporally adjacent precursor samples associated with a given attack episode remain within the same simulation-run partition, overlapping 5 s warning windows from one event cannot be divided across training, validation, and testing subsets. This prevents closely correlated precursor observations belonging to the same attack evolution from producing cross-partition leakage.

Nevertheless, the training, validation, and testing realizations share the same reference topology, network-component counts, radio settings, mobility-category distributions, traffic-generation assumptions, handover configuration, and IRDA attack-generation framework. Therefore, the run-disjoint evaluation specifically measures within-configuration stochastic generalization and should not be interpreted as evidence of generalization across substantially different TN–NTN deployments. In particular, transfer performance under different satellite constellation sizes, UE densities, gateway placements, terrestrial access layouts, mobility distributions, traffic-generation settings, and independently designed attack scenarios has not been established by the present experimental protocol. Demonstrating such capabilities requires additional experiments using explicitly separated network configurations or topology-level holdout scenarios.

### 3.3. Proposed Handover-Aware Temporal Graph Learning Framework for IRDA Classification and Prediction

The proposed Handover-Aware Temporal Graph Transformer (HATGT) aims to extract the spatial and temporal dependencies that exist between radio conditions, mobility evolution, handover progression, session state, and forwarding behavior in the TN–NTN environment. HATGT first transforms each network observation into a heterogeneous relational graph, and then models the evolution of these graphs across successive time steps, rather than using the traditional models that use independent feature vectors for each network observation. This design is inspired by the structure of IRDA itself: a serving satellite, gateway, route version or session state may be valid alone, but the temporal relationship may be inconsistent among the four. The proposed framework is thus not about finding a single abnormal variable, but rather, it examines whether the radio/mobility state, handover state, session state and routing state change in an appropriate way during the transition between TN–TN, TN–NTN, NTN–TN, and NTN–NTN. The entire process flow is as follows:

$$\begin{array}{c} \mathrm{Temporal}\;\mathrm{observations}\to \mathrm{heterogeneous}\;\mathrm{graph}\;\mathrm{construction}\to \\ \;\mathrm{relation}-\mathrm{aware}\;\mathrm{graph}\;\mathrm{encoding}\to \mathrm{handover}-\mathrm{aware}\;\mathrm{encoding}\to \\ \mathrm{temporal}\;\mathrm{Transformer}\to \mathrm{contrastive}\;\mathrm{representation}\;\mathrm{learning}\to \\ \mathrm{multi}-\mathrm{task}\;\mathrm{prediction}. \end{array}$$

The resulting representation supports four complementary security objectives derived from the targets introduced in Section 3.2: six-class IRDA classification, binary IRDA detection, 5 s early-warning prediction, and time-to-IRDA estimation.

The proposed model removes any direct attack targets and metadata variables required for reproducibility/repartitioning, such as run_id and seed, and does not guarantee the absence of simulation-specific or attack-generating-specific correlations in the remaining features. In particular, although some continuous measurements are missing mismatch indicators, the measurements of radio, mobility, handover, routing, traffic, QoS, and session are estimated using statistics derived only from the training partition, while the categorical network entities are embedded into the trainable latent representations. It then maps each UE centered observation with its serving and candidate access nodes, forwarding anchors, UPF and application side connectivity. This representation is meant to enable discrimination between changes in the physical access topology, and the corresponding changes to the control and data-planes, and between legitimate handover recovery, and delayed-route attacks, or between legitimate gateway transitions, and gateway-binding inconsistencies, with the use of temporally related measures. It is, however, a modeling hypothesis that is assessed in the current configuration of the simulator and does not necessarily imply that decisions are exclusively based upon IRDA evidence that are mechanism-invariant.

#### 3.3.1. Temporal Heterogeneous Graph Construction

At simulation time *t*, the TN–NTN state is represented as a heterogeneous graph

$$G_{t}=\left(V_{t},E_{t},X_{t}\right),$$

where $V_{t}$ denotes the set of network entities, $E_{t}$ denotes typed relationships among these entities, and $X_{t}$ represents the associated node and edge attributes. The node set is defined as

$$V_{t}=V^{UE}\cup V^{gNB}\cup V^{SAT}\cup V^{GW}\cup V^{UPF}\cup V^{EDGE}.$$

Thus, the graph explicitly preserves the heterogeneous semantics of UEs, terrestrial gNBs, LEO satellites, NTN gateways, the UPF, and edge/application servers rather than treating all network entities as indistinguishable vertices. Representative relation types include

$$UE\overset{\mathrm{SERVED}\_\mathrm{BY}}{\to }gNB/SAT,$$

$$UE\overset{\mathrm{CANDIDATE}\_\mathrm{ACCESS}}{\to }gNB/SAT,$$

$$SAT\overset{\mathrm{RELAYED}\_\mathrm{BY}}{\to }GW,$$

$$GW\overset{\mathrm{FORWARDED}\_\mathrm{TO}}{\to }UPF,$$

$$UPF\overset{\mathrm{CONNECTED}\_\mathrm{TO}}{\to }EDGE,$$

and

$$UE\overset{\mathrm{HANDOVER}\_\mathrm{TO}}{\to }gNB/SAT.$$

The serving and candidate relations are dynamically reconstructed at each observation instant from the dataset fields serving_node_id, candidate_node_id, serving_node_type, and candidate_node_type. Control-plane and data-plane associations are incorporated through control_anchor_id and data_anchor_id, allowing the graph to preserve the two states separately rather than collapsing them into a manually generated mismatch indicator.

For each UE *u*, the initial dynamic feature vector is represented as

$$x_{u}^{t}=\left[x_{u,t}^{radio}∥x_{u,t}^{mob}∥x_{u,t}^{ho}∥x_{u,t}^{route}∥x_{u,t}^{qos}∥x_{u,t}^{session}\right],$$

where ∥ denotes vector concatenation. The six feature groups correspond respectively to radio/NTN measurements, mobility characteristics, handover evolution, routing state, traffic/QoS measurements, and session context. This decomposition reflects the cross-domain nature of IRDA. Semantically motivated feature grouping cannot rule out shortcut correlations between those features and the simulation or attack-generation procedures.

To preserve temporal continuity during heterogeneous graph learning, the observation sequence is reconstructed from the synchronized simulation records before class-aware selection of learning windows. Specifically, observations are grouped by the unique combination of run_id and ue_id, chronologically ordered according to timestamp_s, and verified against the nominal sampling interval of Δt=0.5 s. For each monitored UE, the temporal representation consists of L=20 consecutive graph snapshots, corresponding to 20 observation instants of historical context, with a 9.5 s elapsed time between the first and final observation. The resulting UE-centered temporal graph sequence is defined as

$$W_{r,u,t}=\left[G_{r,u,t-(L-1)\Delta t},\ldots ,G_{r,u,t-\Delta t},G_{r,u,t}\right],\;L=20,\;\Delta t=0.5\;s.$$

A candidate window is considered temporally valid only when all *L* observations belong to the same simulation run and UE trajectory and every pair of adjacent timestamps satisfies $t_{i+1}-t_{i}=0.5$ s. Windows containing missing intermediate observations, irregular sampling gaps, or incomplete historical context are excluded rather than being artificially reconstructed through interpolation or padding. This criterion ensures that the graph encoder receives physically consecutive network states rather than unrelated observations that become adjacent solely after sampling.

Class-aware sampling is applied to eligible complete temporal windows according to the target label associated with each window’s final observation, rather than independently removing intermediate timestamps from the underlying UE trajectory. Consequently, class balancing affects the selection frequency of complete learning sequences without changing their internal temporal order, sampling resolution, or graph-state transitions. Historical observations remain available as input context even when they are not independently selected as prediction endpoints. The 60 independent simulation runs are separated into 42 training, nine validation, and nine testing runs, and complete temporal windows are generated within their corresponding partitions.

#### 3.3.2. Relation-Aware Graph Encoder

Spatial dependencies within each graph snapshot are learned using a relation-aware heterogeneous attention encoder. For node *v*, the representation at graph layer l+1 is computed as

$$h_{v}^{\left(l+1\right)}=\sigma \left(W_{0}^{\left(l\right)}h_{v}^{\left(l\right)}+\sum_{r\in R}\sum_{u\in N_{r}\left(v\right)}\alpha_{uv}^{r,l}W_{r}^{\left(l\right)}h_{u}^{\left(l\right)}\right),$$

where R is the set of graph relation types, $N_{r}\left(v\right)$ is the set of neighbors connected to *v* through relation *r*, $W_{r}^{\left(l\right)}$ is a relation-specific projection matrix, and $\alpha_{uv}^{r,l}$ is the attention coefficient assigned to neighbor *u*. The attention score is obtained as

$$e_{uv}^{r,l}=\frac{{\left(W_{Q}^{r}h_{v}^{\left(l\right)}\right)}^{⊤}\left(W_{K}^{r}h_{u}^{\left(l\right)}\right)}{\sqrt{d_{k}}},$$

followed by relation-specific normalization,

$$\alpha_{uv}^{r,l}=\frac{\exp(e_{uv}^{r,l})}{\sum_{j\in N_{r}\left(v\right)}\exp\left(e_{jv}^{r,l}\right)}.$$

This formulation permits the same neighboring entity to have different significance depending on the relation through which it interacts with the UE. For example, a satellite acting as the current serving node should influence the UE representation differently from the same satellite appearing only as a candidate access node. Similarly, a gateway involved in the current forwarding path provides different security information from a gateway associated only with the control state.

#### 3.3.3. Handover-Aware State Encoding

A central component of HATGT is the explicit handover-aware representation that informs the temporal model about the expected progression of legitimate mobility transitions. For UE *u* at time *t*, the handover descriptor is defined as

$$q_{u,t}^{HO}=\left[e_{phase},e_{type},a_{u,t}^{A3},p_{u,t}^{TTT},T_{u}^{TTT},\tau_{u,t}^{lastHO},d_{u,t}^{lastHO},c_{u,t}^{HO},r_{u,t}^{age},e_{session}\right],$$

where $e_{phase}$ and $e_{type}$ denote embeddings of the handover phase and transition type, $a_{u,t}^{A3}$ is the A3 margin, $p_{u,t}^{TTT}$ represents TTT progress, $T_{u}^{TTT}$ is the configured TTT, $\tau_{u,t}^{lastHO}$ denotes the time elapsed since the previous handover, $d_{u,t}^{lastHO}$ denotes its duration, $c_{u,t}^{HO}$ is the accumulated handover count, and $r_{u,t}^{age}$ represents the age of the active route.

The handover state is projected into the same latent space as the graph representation:

$$h_{u,t}^{HO}=\phi \left(W_{HO}q_{u,t}^{HO}+b_{HO}\right),$$

and fused with the graph embedding according to

$${\tilde{z}}_{u,t}=z_{u,t}^{G}+\gamma_{u,t}⊙h_{u,t}^{HO},$$

where $z_{u,t}^{G}$ is the relation-aware graph representation and $\gamma_{u,t}$ is a learnable gating vector,

$$\gamma_{u,t}=\sigma \left(W_{g}\left[z_{u,t}^{G}∥h_{u,t}^{HO}\right]+b_{g}\right).$$

The gating mechanism allows the network to increase the influence of handover information during PREPARATION, EXECUTION, or RECOVERY while reducing unnecessary handover emphasis during long STABLE periods. This mechanism distinguishes HATGT from a generic Temporal Graph Transformer by conditioning the fused representation on mobility context. The learned gate is not constrained to isolate causal mobility evidence; therefore, its behavior should be assessed through feature and component ablations, with the interpretation limited to predictive contribution within the evaluated distribution.

#### 3.3.4. Handover-Aware Temporal Graph Transformer

The Handover-Aware Temporal Graph Transformer (HATGT) learns temporal dependencies among the heterogeneous network-state representations obtained from the relation-aware graph encoder and the handover-aware fusion mechanism. The architectural contribution is therefore associated with how the available observations are integrated and processed across network entities and successive time instants, rather than with the mere availability of attack-relevant features. In particular, anchor, routing, handover, and session-state attributes provide informative measurements of the cross-layer conditions associated with IRDA, whereas the temporal Transformer is designed to learn their evolving relationships and distinguish legitimate mobility-induced transitions from potentially malicious state divergence.

For a UE-centered temporal window of length *L*, the sequence passed to the temporal encoder is

$$Z_{u,t-L+1:t}=\left[{\tilde{z}}_{u,t-L+1},\ldots ,{\tilde{z}}_{u,t}\right].$$

A learnable temporal positional embedding $p_{k}$ is added to each state,

$$s_{u,k}={\tilde{z}}_{u,k}+p_{k}.$$

For attention head *m*, the temporal attention mechanism computes

$$Q_{m}=SW_{m}^{Q},\;K_{m}=SW_{m}^{K},\;V_{m}=SW_{m}^{V},$$

and

$${\mathrm{Attn}}_{m}=\mathrm{softmax}\left(\frac{Q_{m}K_{m}^{⊤}}{\sqrt{d_{k}}}+M\right)V_{m},$$

where M is a causal mask preventing information from future observations from contributing to the current prediction. Multi-head outputs are concatenated and processed through residual connections, layer normalization, and feed-forward transformations. After $N_{T}$ temporal layers, the final UE representation is

$$h_{u,t}^{*}=f_{\mathrm{HATGT}}\left(G_{t-L+1:t},q_{u,t-L+1:t}^{HO}\right).$$

The principal dependency learned by this representation can be summarized as

Radio/Mobility↔Handover↔Session↔Routing.

The resulting representation integrates two complementary sources of predictive information. The first is the discriminative content of the network-state attributes extracted from the simulated TN–NTN environment, including serving and candidate access measurements, handover progression, control-plane and data-plane anchor associations, routing evolution, and session-state transitions. These features are intentionally related to the physical and logical processes involved in IRDA and may provide substantial predictive information even when processed by simpler learning methods. The second source is the HATGT architecture, which combines relation-aware graph aggregation, handover-conditioned representation fusion, and causal temporal attention to model relationships among these observations across network entities and time. Consequently, the architectural contribution should be interpreted as improved learning from the available cross-layer telemetry rather than as evidence that equivalent performance could be achieved independently of the underlying feature design.

The distinction is particularly relevant to early-warning prediction and time-to-IRDA estimation. Current-state classification may benefit directly from informative routing, anchor, and session-state measurements, whereas forecasting a future IRDA onset additionally requires the interpretation of how these states evolve before attack activation. Causal temporal attention enables the model to combine previously observed network states without accessing future observations, while relation-aware graph encoding represents the heterogeneous associations through which mobility and forwarding changes are reflected. The handover-aware encoder and adaptive fusion mechanism further condition these representations on legitimate handover progression. These architectural mechanisms are intended to improve the interpretation of temporal and relational dependencies, but their individual contributions must be assessed through controlled ablation experiments rather than inferred solely from their mathematical formulation.

The intended detection signal is a learned departure from the temporal relationships observed among radio/mobility, handover, session, and routing states. Because the model is optimized using simulated observations and attack labels, successful discrimination does not prove that the learned dependencies are invariant to alternative attack implementations or traffic and mobility distributions. Moreover, performance differences obtained by removing an architectural component while retaining the original feature set quantify architecture sensitivity under that feature configuration, whereas removing feature groups while retaining the complete architecture quantifies dependence on the available network-state information. These two forms of ablation provide complementary evidence but do not uniquely separate architectural and feature-design contributions because the corresponding effects may interact. A more direct assessment requires evaluating HATGT and alternative learning architectures using identical full and reduced feature sets under the same run-disjoint experimental protocol.

#### 3.3.5. Supervised Contrastive Representation Learning

A supervised contrastive objective is incorporated to improve separation among NORMAL behavior and the five IRDA mechanisms while preserving the natural variability of legitimate mobility transitions. Let $r_{i}$ denote the normalized projection of the final temporal representation for sample *i*,

$$r_{i}=\frac{g(h_{i}^{*})}{∥g\left(h_{i}^{*}\right){∥}_{2}}.$$

The supervised contrastive loss is defined as

$$L_{con}=-\sum_{i}\frac{1}{\left|P(i)\right|}\sum_{p\in P(i)}\log\frac{\exp\left(r_{i}^{⊤}r_{p}/\tau \right)}{\sum_{a\neq i}\exp\left(r_{i}^{⊤}r_{a}/\tau \right)},$$

where P(i) denotes samples sharing the same multiclass attack label as sample *i*, and τ denotes the contrastive temperature. The objective encourages samples from the same IRDA mechanism to occupy nearby regions in latent space while separating representations corresponding to different attack mechanisms. Because the contrastive positives and negatives are defined using labels from the same attack-generation process, this objective does not independently prevent the model from learning generator-associated regularities.

#### 3.3.6. Multi-Task Security Prediction Heads

The shared HATGT representation is connected to four task-specific prediction heads. The first head performs six-class IRDA classification:

$${\hat{y}}_{u,t}^{cls}=\mathrm{softmax}\left(W_{cls}h_{u,t}^{*}+b_{cls}\right),$$

where the output classes are

{NORMAL,IRDA-1,IRDA-2,IRDA-3,IRDA-4,IRDA-5}.

The second head performs binary current-state detection,

$${\hat{y}}_{u,t}^{bin}=\sigma \left(W_{bin}h_{u,t}^{*}+b_{bin}\right),$$

corresponding to target_binary_irda.

The third head estimates the probability that an IRDA onset will occur within the subsequent 5 s,

$${\hat{y}}_{u,t}^{future}=\sigma \left(W_{pred}h_{u,t}^{*}+b_{pred}\right),$$

corresponding to target_future_irda_5s. Because only 1.718% of the observations belong to the positive forecasting class, class weighting is applied during optimization so that failure to detect rare pre-attack states is not masked by the dominant negative class.

Finally, the regression head estimates the remaining time to the next IRDA event,

$${\hat{T}}_{u,t}=\mathrm{Softplus}\left(W_{reg}h_{u,t}^{*}+b_{reg}\right),$$

where the non-negative output corresponds to target_time_to_irda_ms. A logarithmic transformation,

$${\tilde{T}}_{u,t}=\log\left(1+T_{u,t}\right),$$

is used during optimization to reduce the numerical dominance of long time-to-event intervals.

#### 3.3.7. Joint Optimization Objective

The final HATGT model is trained through a weighted multi-task objective:

$$L_{total}=\lambda_{cls}L_{cls}+\lambda_{bin}L_{bin}+\lambda_{pred}L_{pred}+\lambda_{reg}L_{reg}+\lambda_{con}L_{con},$$

where $L_{cls}$ is the six-class cross-entropy loss, $L_{bin}$ is the binary current-state detection loss, $L_{pred}$ is the class-weighted future-IRDA prediction loss, $L_{reg}$ is the Smooth-$L_{1}$ time-to-event regression loss, and $L_{con}$ is the supervised contrastive loss. The coefficients $\lambda_{cls}$, $\lambda_{bin}$, $\lambda_{pred}$, $\lambda_{reg}$, and $\lambda_{con}$ control the relative contribution of each objective. However, multi-task optimization does not guarantee against shortcut learning, since no matter which objective is learned, the underlying assumptions of the simulator and attack generator are shared.

  Scope of shortcut-learning safeguards. The exclusions outlined in the predictive-input, the run-disjoint partitions, the temporal causal mask, heterogeneous relation modeling, and the  handover-aware fusion all capture identifiable leakage channels, or provide useful inductive biases, without any being a formal shortcut-elimination guarantee. The performance changes caused by ablation may exhibit group or architectural block dependence, but this does not prove that either dependence is causal or it is physically transferrable or that the dependence is correlated with artifacts of the simulator. The same reference topology and prescribed traffic, mobility, handover and IRDA-generation mechanisms are applied to all 60 runs of the datasets. To directly test reliance on shortcuts would necessitate the evaluation of frozen model checkpoints on controlled distribution shifts (e.g., varying attack timing and intensity, other mobility generators, other traffic generators, and altered satellite/gateway configurations), with outcomes of the attacks re-labeled after the new simulation traces. The present evaluation does not involve these experiments, so no claims are made regarding shortcut-free representations and cross-configuration generalization.  

**Figure 3:**
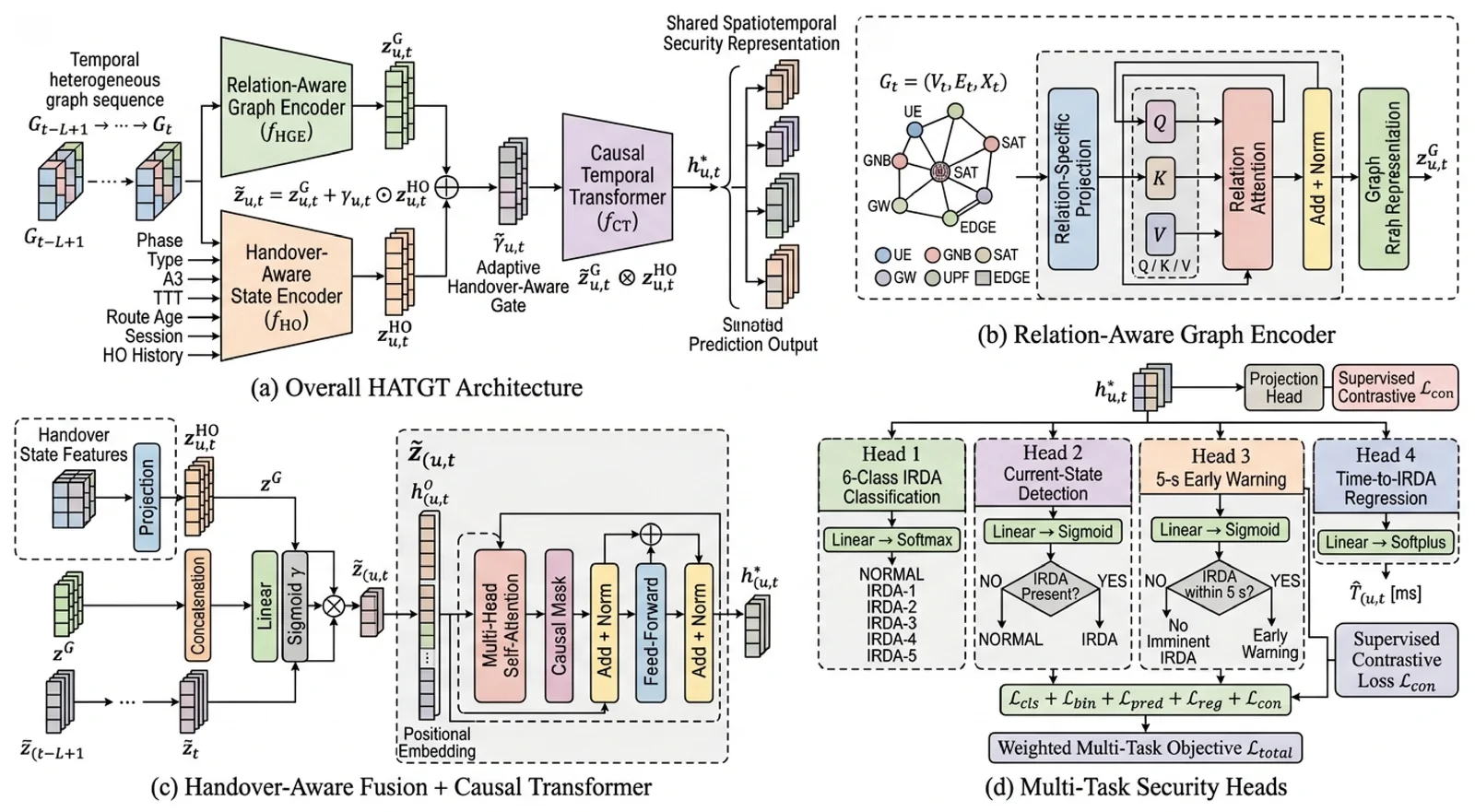
Architecture of the proposed Handover-Aware Temporal Graph Transformer.

Figure 3 illustrates the internal organization of the proposed HATGT as a unified spatiotemporal security architecture that progressively transforms heterogeneous graph states into temporally consistent multi-task security decisions. In Figure 3a, the relation-aware graph encoder $f_{\mathrm{HGE}}$ extracts topology-dependent representations $z_{u,t}^{G}$, while the parallel handover-aware encoder $f_{\mathrm{HO}}$ captures mobility-transition evidence from phase, transition type, A3 margin, TTT progression, route age, session state, and recent handover history. These complementary representations are combined through the adaptive gate $\gamma_{u,t}$, allowing the model to regulate the contribution of handover information before producing the fused state ${\tilde{z}}_{u,t}$. Panel (b) further exposes the relation-aware mechanism, where heterogeneous node interactions are projected into relation-specific query, key, and value representations and processed by attention and residual normalization, enabling identical network entities to receive different importance according to their relational roles. Panel (c) then models the sequence ${\tilde{z}}_{u,t-L+1:t}$ using positional embeddings, causal multi-head self-attention, masking, residual normalization, and feed-forward transformations, yielding the shared security representation $h_{u,t}^{*}$ without accessing future states. Finally, panel (d) distributes this representation across four coordinated heads for six-class IRDA attribution, current-state NORMAL/IRDA detection, 5 s early warning, and non-negative time-to-IRDA regression. The auxiliary supervised contrastive objective $L_{con}$, together with $L_{cls}$, $L_{bin}$, $L_{pred}$, and $L_{reg}$, forms the weighted objective $L_{total}$, forcing HATGT to jointly preserve relational structure, mobility context, temporal causality, class separability, and attack-proximity information within a single learned representation.

Algorithm 1 formalizes the complete optimization process of the proposed HATGT by integrating leakage-safe data preparation, heterogeneous graph reasoning, explicit handover modeling, temporal attention, and multi-task security learning within a single end-to-end procedure. The algorithm begins by enforcing run-disjoint training, validation, and testing partitions before any feature normalization or categorical transformation is performed, thereby preventing training-time preprocessing statistics from being estimated using held-out simulation runs. Run-disjoint evaluation addresses within-run contamination, but the shared simulator, traffic models, and attack generator may still create correlations common to both training and test runs. Within each UE-specific temporal window, the network state at every observation instant is reconstructed as a heterogeneous TN–NTN graph containing the UE and its dynamically associated serving access node, candidate access node, gateway/anchor state, UPF, and edge-side connectivity. The relation-aware graph encoder $f_{\mathrm{HGE}}$ subsequently extracts spatial representations that preserve the semantic differences among serving, candidate, handover, gateway, and forwarding relations instead of treating all neighboring entities uniformly. In parallel, $f_{\mathrm{HO}}$ encodes the mobility-transition context using handover phase, handover type, A3 margin, TTT progression, route age, session state, and recent handover history. The adaptive gating vector $\gamma_{u,k}$ then controls the contribution of handover information to the spatial graph state, allowing the model to place stronger emphasis on mobility context during PREPARATION, EXECUTION, and RECOVERY while avoiding unnecessary sensitivity during stable operation. The sequence of fused graph states is processed by the causal temporal encoder $f_{\mathrm{CT}}$, which learns how radio, mobility, handover, session, and routing states evolve jointly without accessing future observations. The final latent representation is shared across multiclass classification, binary detection, 5 s early-warning prediction, and time-to-IRDA regression, while supervised contrastive learning improves separation between NORMAL behavior and the five IRDA mechanisms. Consequently, the joint objective does not optimize HATGT for a single detection output; instead, it forces the shared representation to simultaneously encode current attack state, attack mechanism, precursor behavior, and temporal proximity to an upcoming IRDA event.

- The run-disjoint preprocessing stage preserves experimental independence by ensuring that observations originating from the same simulation realization cannot be distributed across training, validation, and testing subsets. Numerical normalization and categorical encoding are fitted exclusively on the training runs, preventing preprocessing-level information leakage and providing an estimate of generalization to unseen stochastic runs under the same reference TN–NTN configuration, not to unseen network configurations or independently generated attacks.
- The heterogeneous graph construction stage preserves the physical and logical organization of the integrated network by explicitly representing UE, gNB, satellite, gateway, UPF, and edge entities together with relation-specific connectivity. Dynamic SERVED_BY, CANDIDATE_ACCESS, handover, gateway, and forwarding relationships allow IRDA to be modeled as an inconsistency among interacting network states rather than as an isolated abnormal feature value.
- The relation-aware encoder $f_{\mathrm{HGE}}$ assigns different transformations and attention importance to different graph relations. This is essential because a satellite acting as the current serving node has a different security meaning from the same satellite acting only as a candidate node, while a gateway participating in the active data path carries different information from a gateway associated with the control context.

**Algorithm 1** Training of the proposed Handover-Aware Temporal Graph Transformer.**Require:** TN–NTN dataset D; temporal length *L*; epochs *E*; batch size *B*; learning rate η**Ensure:** Optimized HATGT parameters $\Theta^{*}$  1:Partition D into run-disjoint $D_{tr}$, $D_{val}$, and $D_{te}$  2:Remove identifiers, targets, and leakage-prone variables from predictive inputs  3:Fit numerical normalization and categorical encoding using $D_{tr}$ only  4:Group records by (run_id,ue_id) and sort by timestamp_s  5:Construct temporal windows $S_{u,t}=\left\{x_{u,t-L+1},\ldots ,x_{u,t}\right\}$  6:Initialize model parameters Θ  7:**for** e=1 to *E* **do**  8:      **for** each mini-batch $B\subset D_{tr}$ **do**  9:            **for** each $S_{u,t}\in B$ **do**10:                  **for** k=t−L+1 to *t* **do**11:                        Construct heterogeneous TN–NTN graph $G_{k}=\left(V_{k},E_{k},X_{k}\right)$12:                        $z_{u,k}^{G}\leftarrow f_{\mathrm{HGE}}\left(G_{k}\right)$▹ relation-aware spatial encoding13:                        $z_{u,k}^{HO}\leftarrow f_{\mathrm{HO}}\left(q_{u,k}^{HO}\right)$▹ handover-state encoding14:                        $\gamma_{u,k}\leftarrow \sigma \;\left(W_{g}\left[z_{u,k}^{G}∥z_{u,k}^{HO}\right]+b_{g}\right)$15:                        ${\tilde{z}}_{u,k}\leftarrow z_{u,k}^{G}+\gamma_{u,k}⊙z_{u,k}^{HO}$16:                  **end for**17:                  $h_{u,t}\leftarrow f_{\mathrm{CT}}\left({\tilde{z}}_{u,t-L+1:t}\right)$▹ causal temporal encoding18:                  ${\hat{y}}^{cls}\leftarrow f_{cls}\left(h_{u,t}\right)$; ${\hat{y}}^{bin}\leftarrow f_{bin}\left(h_{u,t}\right)$19:                  ${\hat{y}}^{future}\leftarrow f_{pred}\left(h_{u,t}\right)$; $\hat{T}\leftarrow f_{reg}\left(h_{u,t}\right)$20:            **end for**21:            Compute $L_{cls}$, $L_{bin}$, $L_{pred}$, $L_{reg}$, and  $L_{con}$22:            $L_{total}\leftarrow \lambda_{1}L_{cls}+\lambda_{2}L_{bin}+\lambda_{3}L_{pred}+\lambda_{4}L_{reg}+\lambda_{5}L_{con}$23:            Update Θ using AdamW: $\Theta \leftarrow \Theta -\eta \nabla_{\Theta }L_{total}$24:      **end for**25:      Evaluate Θ on $D_{val}$26:      Store $\Theta^{*}$ when validation Macro-F1 improves27:      **if** early-stopping criterion is satisfied **then**28:            **break**29:      **end if**30:
**end for**
31:Evaluate $\Theta^{*}$ on the unseen runs in $D_{te}$32:**return**
 $\Theta^{*}$

- The handover-aware encoder $f_{\mathrm{HO}}$ explicitly incorporates mobility-transition evidence that would otherwise have to be inferred indirectly by a generic graph model. Handover phase, type, A3 margin, TTT progression, route age, session state, and recent transition history jointly indicate whether changes in serving nodes, routing state, or anchor association are expected consequences of mobility or potentially malicious desynchronization.
- The adaptive fusion mechanism ${\tilde{z}}_{u,k}=z_{u,k}^{G}+\gamma_{u,k}⊙z_{u,k}^{HO}$ provides context-dependent control over the contribution of handover information. The learned gate adjusts mobility awareness according to the instantaneous interaction between graph structure and handover progression. Whether it consistently discounts benign transitions outside the simulated training conditions remains untested.
- The causal temporal encoder and joint multi-task objective transform the final representation from a static intrusion descriptor into a temporal security state. Causal attention captures dependencies only from observations available up to the current instant, while the combined classification, binary detection, future-prediction, regression, and contrastive losses train HATGT to distinguish established IRDA activity, identify the attack variant, recognize pre-attack evolution within the 5 s forecasting horizon, and estimate remaining time before attack onset within the constructed benchmark.

Interpretation of feature and component ablations. In the associated ablation analysis, a decrease in classification, detection, or forecasting performance after removing a feature group or module is interpreted as evidence of its predictive contribution under the benchmark sampling and simulation conditions. Such a decrease cannot distinguish physically meaningful dependence from reliance on accidental regularities shared across the train/test simulation runs. Therefore, the ablations support architectural and feature-contribution comparisons, rather than excluding simulator-specific or attack-generator-specific shortcut learning.  

Table 4 sets the complete reproducibility profile of HATGT by providing the organization of temporal data, the heterogeneous graph representation, the optimization strategy and the computational execution in a single configuration. The prediction is made from a series of 20 UE-centered graph snapshots, which give a temporal history of 10 s at a sampling rate of 0.5 s, whereas the evaluation of a forecasting objective is over the next 10 temporal steps of 5 s. The shared 192-dimensional representation is expressive enough to allow for seamless embedding of heterogeneous topology and handover context without significant model growth; two relation-aware graph layers with four heads encode the immediate neighborhood of the network while three causal Transformer layers with six heads and a 384-dimensional feed-forward stage model the temporal context. Experimental independence is achieved by using a strict train/validation/test split of 60/6/7 run levels for the 60 simulation realizations, which reduces any leakage between the different train levels. Optimization is done jointly with AdamW at 2 × 10^−4^, a batch size of 64, 1 × 10^−4^ weight decay, and gradient clipping of 1.0; the higher forecasting-loss contribution, combined with positive-class weight of 57.21, makes up for the significantly more sparse pre-IRDA data. The full model has only 2.84 million parameters and 214 MB inference memory, despite integrating four prediction modalities—a graph, a handover, a temporal, and a contrastive one—with the four prediction components. Measured on the specified RTX 4070 platform, a batch-one inference time of <3.46 ms per UE temporal sequence is shown after 100 warm-up passes for synchronized evaluation over 1000 evaluations. This measurement does not show any preprocessing/dynamic graph-construction costs or demonstrate end-to-end batch-one scalability in online deployment.

## 4. Discussion, Results, and Comparison

The results of the evaluated models are shown in Table 5 and it is observed that as the models move from a static tabular learning to a joint spatial–temporal representation, the IRDA discrimination increases consistently, showing that IRDA discrimination relies heavily on the network relationships and temporal evolution. The Macro-F1 score of Random Forest is 90.61%, and that of XGBoost is 92.50%, both showing that the feature interactions can be useful, but not complete, when considering the instantaneous discrimination. With the incorporation of graph structure, GAT achieves the best Macro-F1 of 94.90%, while recurrent models enhance performance by leveraging sequential behavior. The complementary role of spatial and temporal dependency is seen as further increase of this value to 96.13% with the Temporal GAT. The Temporal Graph Transformer outperforms the baseline with 97.05% Macro-F1 and 0.983 AUPRC. Compared to the best competitor, HATGT has a 0.998 AUROC, 0.992 AUPRC, 98.42% accuracy, and 98.38% Macro-F1, which is 1.33 percentage points higher. This gain shows that the use of relation-aware graph encoding alone is not enough, and that explicit conditioning with knowledge of the handover can give extra information for identifying legitimate mobility evolution from malicious inconsistencies in identity–route.

Table 6 provides a class-level examination of HATGT and shows that its overall performance is not produced by disproportionately strong recognition of only a subset of IRDA mechanisms. NORMAL behavior achieves the highest F1-score of 98.91%, indicating that the framework effectively learns legitimate TN–NTN mobility and handover evolution despite the presence of transient routing and session changes that can resemble desynchronization. Gateway-binding manipulation is also recognized strongly, reaching 98.66% F1 and 0.995 AUPRC, suggesting that inconsistent anchor evolution produces a comparatively distinctive relational pattern. Stale-context replay and delayed route synchronization achieve F1-scores of 98.44% and 98.32%, respectively, while handover-state injection remains similarly well detected at 98.19%. Coordinated low-rate desynchronization is the most challenging mechanism, with an F1-score of 97.77% and AUPRC of 0.985, which is consistent with its weaker distributed temporal signature. Importantly, the narrow range of F1 (1.14% overall) over all six classes shows consistent attack-specific discrimination and not class-dependent performance concentration.

**Figure 4:**
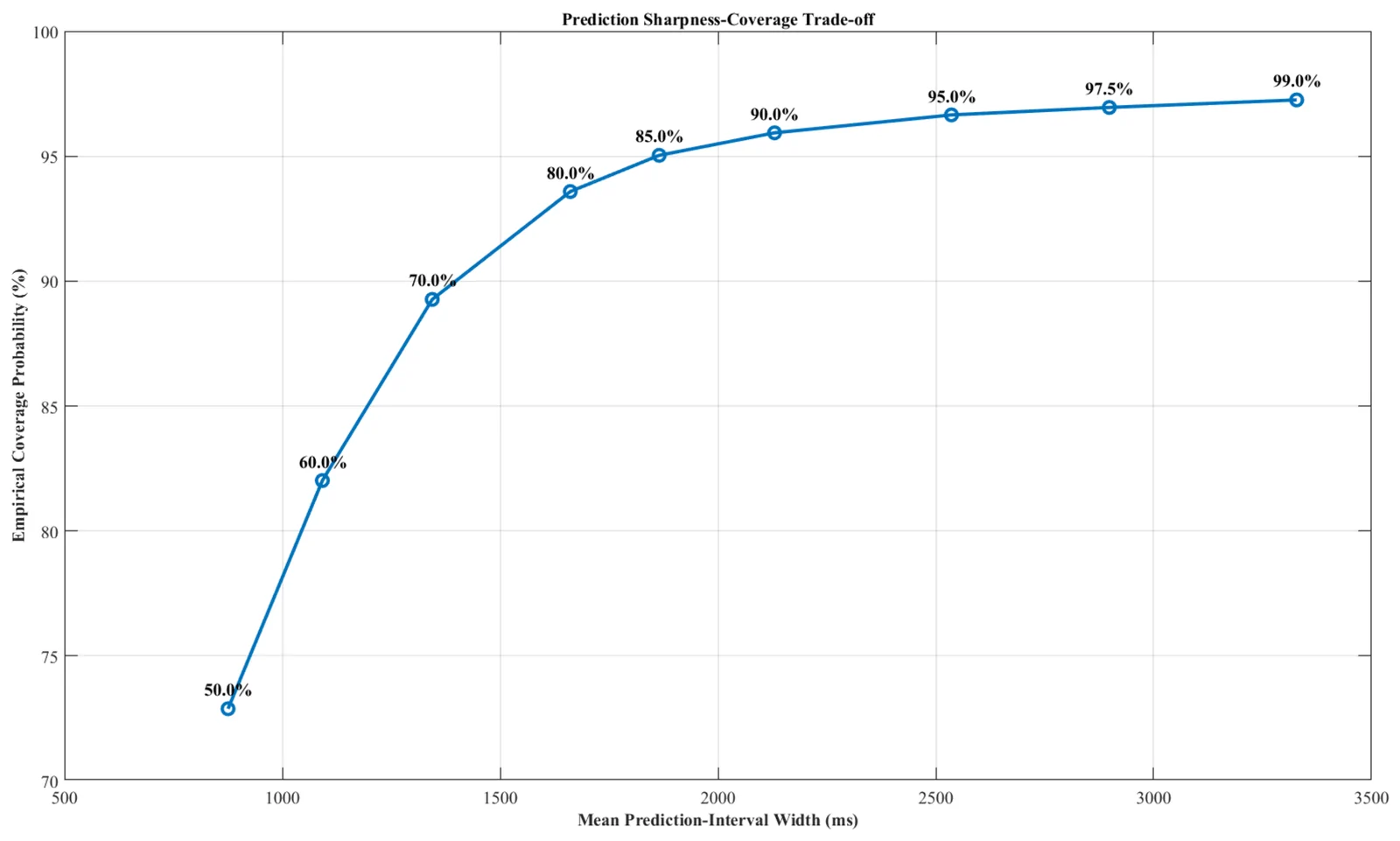
Prediction sharpness–coverage trade-off for the HATGT time-to-IRDA regression head. Prediction intervals are calibrated from validation-set pre-attack residuals and evaluated on unseen test observations with $T_{\mathrm{IRDA}}>0$; marker labels indicate nominal coverage levels, while the vertical axis reports the corresponding empirical test coverage.

The uncertainty–sharpness trade-off of the HATGT time-to-IRDA estimator is characterized in only the valid pre-attack observations with $T_{\mathrm{IRDA}}>0$ (see Figure 4) and excludes the non-countdown targets where $T_{\mathrm{IRDA}}=0$ from the interval analysis. Prediction intervals are calibrated only on the validation runs, with the absolute time-to-event residuals $r_{i}=\left|T_{i}-{\hat{T}}_{i}\right|$. The empirical residual quantile $q_{1-\alpha }$ is estimated from the residual distribution of the validation data for each nominal coverage level 1−α and the test-time interval is set as $\left[\max\left(0,\hat{T}-q_{1-\alpha }\right),\;\hat{T}+q_{1-\alpha }\right]$. The use of test labels is not allowed in calibration during intervals. The percentage above the markers indicates the nominal levels of coverage being investigated (50 to 99 percent) and the vertical axis shows the levels of coverage obtained for unseen pre-attack test samples. The mean interval width ranges from  0.9 s at the lowest operating point up to  3.3 s at the 99% nominal level, while the empirical coverage rises quickly from  73% to >95% and then slowly saturates around 97%. In particular, widths of about 1.3–1.9 s already deliver empirical coverage of close to 89%–95%, while beyond about 2.1 s, the coverage increases less and less. This saturation defines a practically relevant operating range where uncertainty reliability can be improved while maintaining the temporal sharpness within reasonable limits. The analysis thus adds value to the point-estimate MAE and RMSE by providing a quantifiable assessment of the reliability and informativeness of the HATGT time-to-IRDA predictions from true observations of the pre-attack countdown.

Table 7 shows that collapsing the five IRDA mechanisms into a common malicious category produces substantially stronger current-state detection than the more demanding six-class attribution task. The proposed HATGT achieves 98.95% accuracy, 99.64% precision, 99.10% recall, and a 99.37% F1-score, together with AUROC and AUPRC values of 0.999. Although the Temporal Graph Transformer already provides a strong F1-score of 99.13%, HATGT further improves the error characteristics by reducing the false-positive rate from 3.35% to 1.80%, corresponding to an approximate 46% relative reduction, while decreasing the false-negative rate from 1.07% to 0.90%. This behavior is particularly important in the investigated TN–NTN environment because legitimate handovers, route convergence, gateway changes, and session recovery can produce transient conditions resembling malicious desynchronization. However, because the primary binary benchmark contains an 83.33% malicious prevalence by construction, its precision, accuracy, and F1-score characterize discrimination under the controlled experimental distribution rather than expected performance in a low-prevalence operational environment. Table 8 therefore examines the sensitivity of HATGT to substantially lower attack base rates while preserving the fixed operating characteristics of the trained detector. The analysis shows the expected base-rate effect clearly: at 0.1% IRDA prevalence, precision decreases to 5.22% despite maintaining 99.10% recall, whereas at 1%, 5%, and 10% prevalence, it increases to 35.74%, 74.34%, and 85.95%, respectively. The corresponding F1-score rises from 9.92% at 0.1% prevalence to 92.06% at 10%, while the fixed 1.80% false-positive rate produces approximately 179.82 false alarms per 10,000 observations at the lowest prevalence. These results demonstrate why prevalence-sensitive precision and false-alarm burden must accompany accuracy and F1 when interpreting deployment-oriented binary IRDA detection.

**Figure 5:**
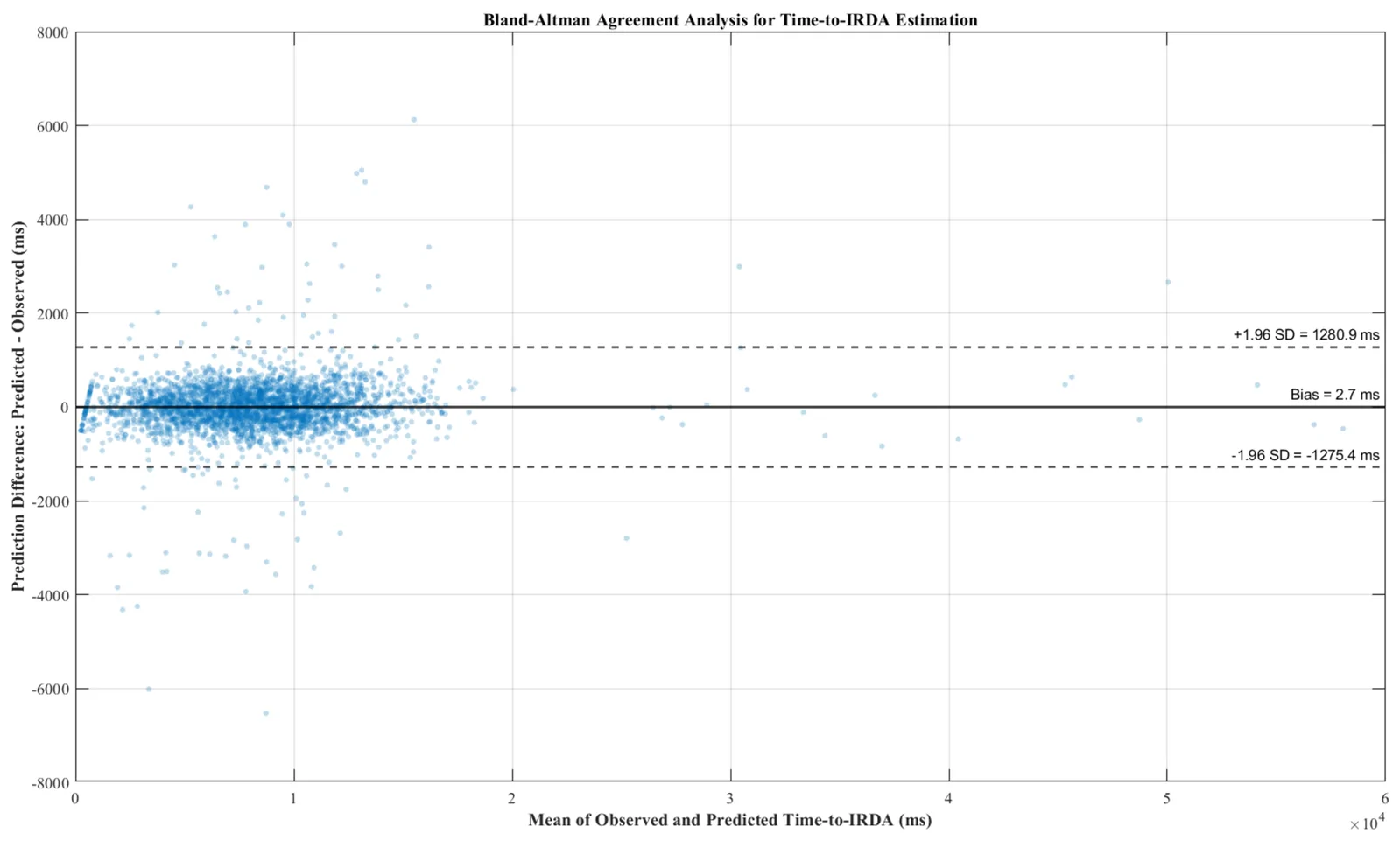
Bland–Altman agreement analysis between observed and HATGT-predicted time-to-IRDA values, including the mean prediction bias and the 95% limits of agreement.

A complementary agreement analysis of the time-to-IRDA regression task is shown as a plot of the differences in predictions versus the mean observed–predicted activation time in Figure 5. The mean bias estimated is 2.7 ms, which suggests that the estimated systematic overestimation or underestimation of the HATGT regression head is practically negligible throughout the range of times evaluated. The limits of agreement for the 95% are approximately −1275.4 to 1280.9 ms with the highest density of observations being very close to the zero error line. This concentration shows that most predictions continue to have good agreement in time even when the actual time-to-IRDA varies greatly. There are some large positive and negative residuals visible, mostly at infrequent long-horizon observations, suggesting that the most challenging forecasting events are in the extreme error tails. Importantly, the lack of obvious monotonic residual trend indicates that prediction bias does not systematically grow with event horizon, consistent with temporal stability of the proposed regression mechanism.

Table 9 represents a more difficult evaluation because the model must identify precursor behavior before an IRDA condition becomes active rather than detect an already established desynchronization state. The performance progression strongly favors models capable of learning temporal context: Random Forest obtains only 59.55% F1 and 0.516 AUPRC, whereas the Temporal Graph Transformer reaches 86.52% F1 and 0.865 AUPRC. HATGT further increases these values to 90.29% and 0.914, respectively, corresponding to a 3.77-percentage-point F1 improvement over the strongest baseline. It also achieves 91.40% precision and 89.20% recall while reducing the false-alarm rate to 0.15% and the miss rate to 10.80%. Compared with the Temporal Graph Transformer, the miss rate decreases by approximately 28%. HATGT additionally provides an average warning lead time of 3.76 s within the defined 5 s horizon, showing that its gain is not limited to classification quality but also provides earlier recognition of developing identity–route inconsistency.

The operational interpretation of the early-warning results requires distinguishing the configured prediction horizon from the actual time available for preventive intervention. The 5 s horizon defines the interval within which an IRDA onset is predicted, whereas the reported 3.76 s represents the average time between the first valid warning and the corresponding attack onset among successfully warned events. Accordingly, the average lead time does not imply that every attack is predicted 3.76 s before activation. Nevertheless, an approximately 3–4 s warning could potentially support preparatory security procedures, such as verifying UE session continuity, checking control-plane and data-plane anchor consistency, validating gateway bindings, examining pending handover transitions, and preparing corrective forwarding-route updates. These operations are relevant to IRDA because the attack mechanisms exploit inconsistencies among mobility, identity/session, gateway, and routing states. Such actions are proposed operational uses of the prediction output and were not implemented or experimentally measured as an integrated mitigation mechanism.

The sufficiency of the available warning interval depends on the complete response time required by the defensive procedure. Let $T_{\mathrm{acq}}$, $T_{\mathrm{prep}}$, $T_{\mathrm{graph}}$, $T_{\inf}$, $T_{\mathrm{decision}}$, $T_{\mathrm{signal}}$, and $T_{\mathrm{mit}}$ denote telemetry acquisition, feature preprocessing, heterogeneous graph construction, model inference, security-decision processing, control-plane signaling, and mitigation execution times, respectively. The total response delay can be expressed as

$$T_{\mathrm{response}}=T_{\mathrm{acq}}+T_{\mathrm{prep}}+T_{\mathrm{graph}}+T_{\inf}+T_{\mathrm{decision}}+T_{\mathrm{signal}}+T_{\mathrm{mit}}.$$

For an early warning associated with attack event *e* to support completion of a preventive action before attack onset, the necessary timing condition is

$$T_{\mathrm{response},e}<\Delta t_{e},$$

where $\Delta t_{e}$ is the warning lead time for that event. The reported average lead time of 3.76 s therefore indicates a potential opportunity for intervention but does not establish that the complete mitigation procedure can be executed within this interval. In particular, the measured 3.46 ms HATGT inference latency represents model-level computation on an NVIDIA RTX 4070 GPU and does not include the complete acquisition, graph-construction, signaling, and mitigation pipeline. Since integrated mitigation delays and the distribution of event-specific response margins were not measured, operational mitigation sufficiency cannot be confirmed from the reported lead time alone.

The early-warning performance must also be interpreted with respect to the selected 5 s prediction horizon. The reported 90.29% F1-score, 0.914 AUPRC, 0.15% false-alarm rate, and 3.76 s average lead time were obtained for this specific forecasting interval and should not be assumed to remain unchanged under alternative horizons. Shorter horizons may emphasize more immediate attack precursors but reduce the available intervention time, whereas longer horizons may increase the potential preparation interval while requiring predictions over more uncertain future network states. Changes in the horizon can also modify positive-label prevalence, the number of precursor observations associated with each attack event, warning lead time, and the precision–recall trade-off. Because separate evaluations using shorter and longer forecasting horizons have not been established in the present experiments, the sensitivity of HATGT performance to horizon selection remains an open question. Consequently, the reported results support simulation-level early-warning capability under the investigated 5 s horizon rather than verified horizon-independent forecasting performance or closed-loop attack-mitigation effectiveness.

The lead-time values in Table 9 describe the advance timing of valid early-warning predictions relative to the corresponding simulated IRDA onsets under the fixed 5 s forecasting horizon. They are predictive performance measures rather than measured intervals of successful attack prevention.

**Figure 6:**
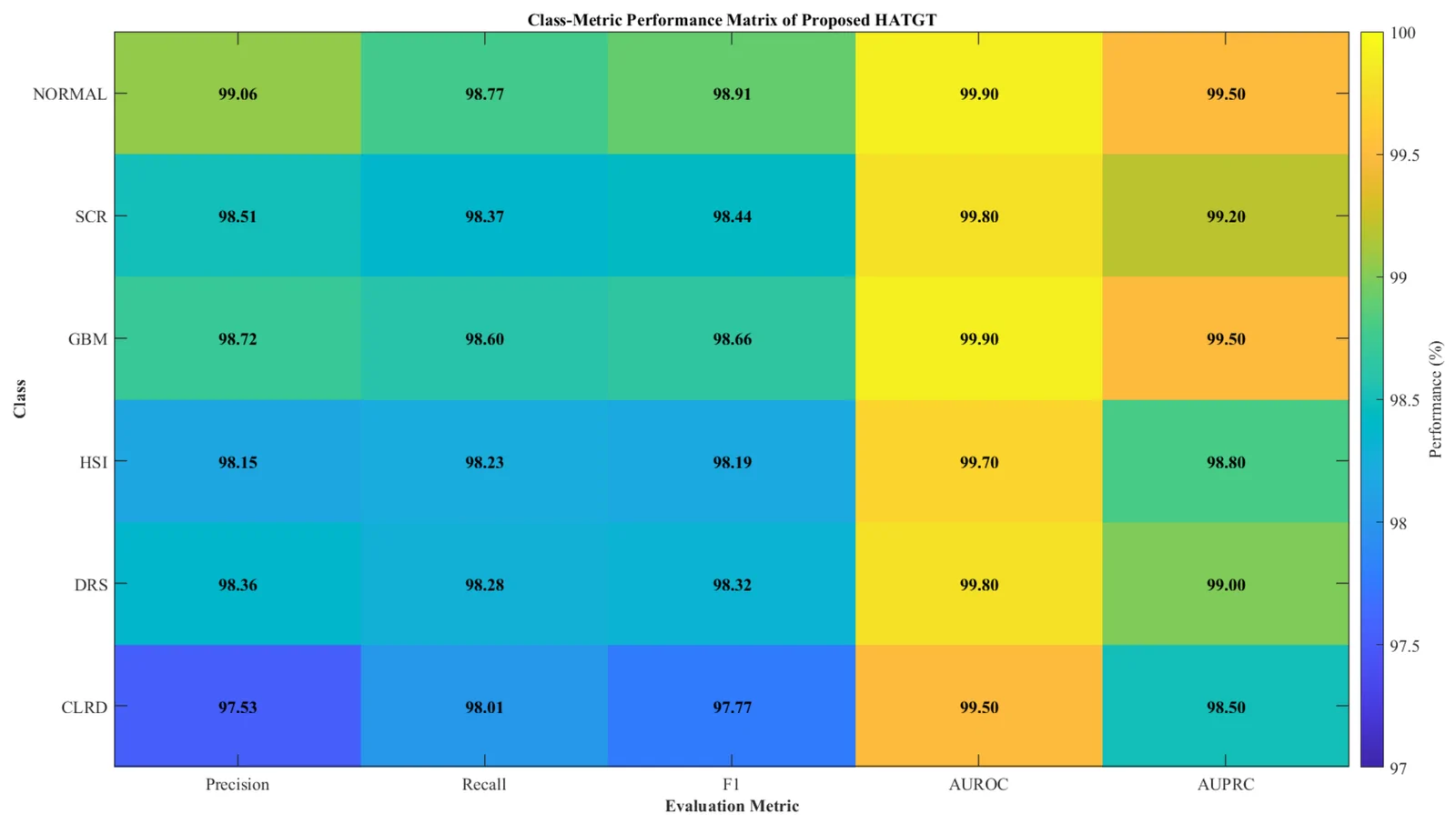
Class–metric performance matrix of the proposed HATGT across NORMAL traffic and five Identity–Route Desynchronization Attack mechanisms.

Figure 6 shows a class-wise assessment of HATGT based on precision, recall, F1-score, AUROC and AUPRC, highlighting excellent discrimination with all the IRDA mechanisms, not by any one attack category. The precision, recall and F1 of NORMAL traffic are 99.06%, 98.77%, and 98.91% respectively, showing that it is successful in rejecting legitimate handover and route-transition behavior. The separation of the Gateway-binding manipulation is also impressive, with a combined F1-score of 98.66% as well as a AUROC of 99.90% and an AUPRC of 99.50%. On the contrary, coordinated low-rate desynchronization is the most complex class, whose accuracy reduces to 97.53% for precision and 97.77% for F1. This decrease is predicted, as it is expected that low-rate desynchronization will lead to more distributed and less strong identity–route inconsistencies than abrupt manipulation mechanisms. Nevertheless, its AUROC of 99.50% and AUPRC of 98.50% remain high. The small gap between the inter-class performance shows that HATGT is not merely using easily distinguishable IRDA signatures, but has a strong detection capability across heterogeneous attack dynamics.

**Figure 7:**
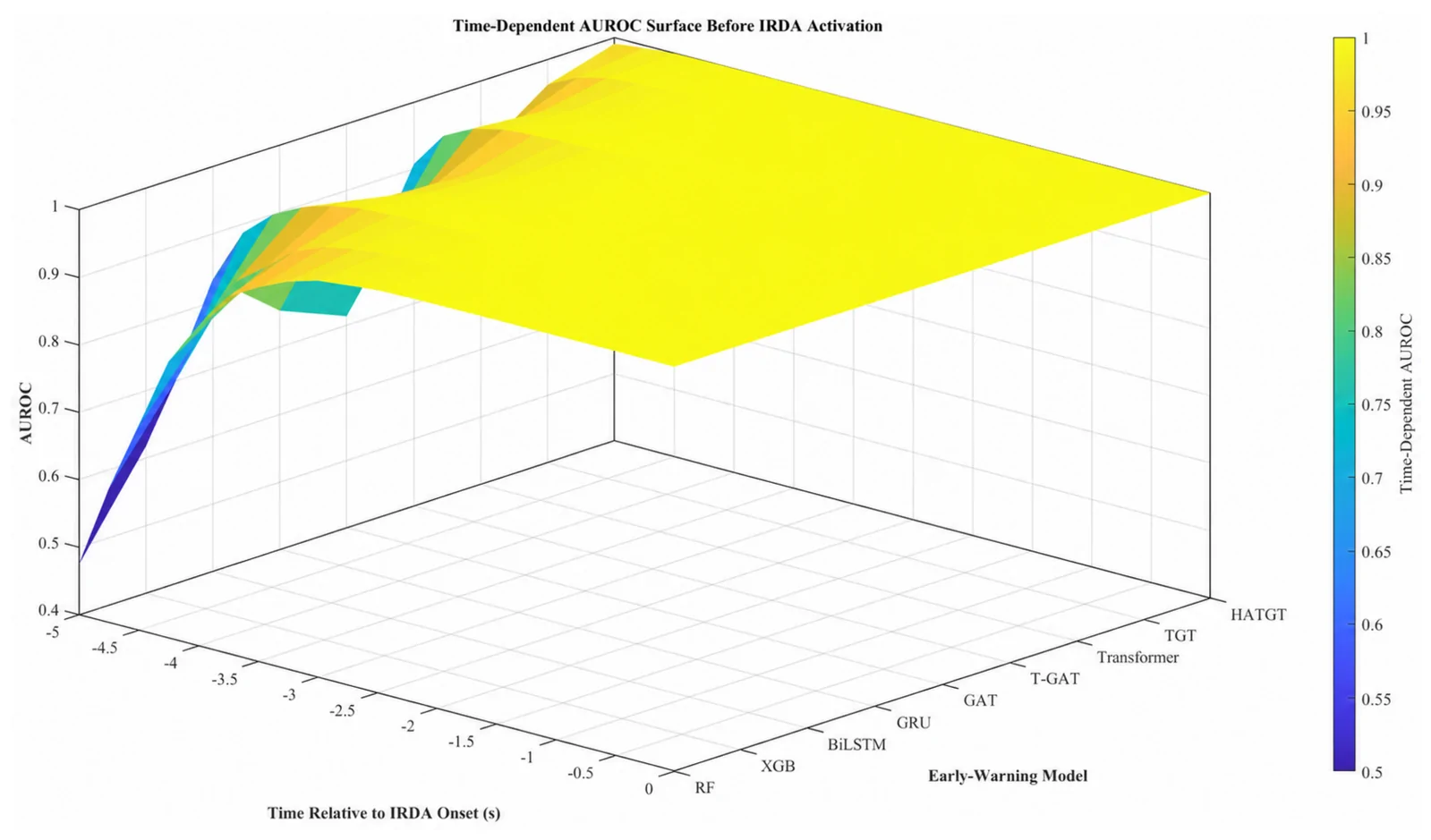
Time-dependent AUROC surface of competing early-warning models as observations progress from five seconds before IRDA onset toward the attack activation time.

Figure 7 extends conventional AUROC evaluation by resolving discrimination capability over time relative to IRDA onset. All models improve as the observation approaches t=0, reflecting the increasing availability of identity–route inconsistency evidence immediately before attack activation. The differences are considerably more informative at the earliest observation region around t=−5 s, where weaker classifiers exhibit substantially reduced separability because only subtle precursor signals are available. Temporal and graph-aware architectures retain stronger discrimination in this difficult region, while HATGT occupies the upper performance surface and approaches near-perfect AUROC earlier than conventional alternatives. This behavior supports the architectural motivation for combining heterogeneous relational encoding with handover-aware state representations and causal temporal attention. Rather than depending only on obvious post-divergence signatures, HATGT can integrate weak radio, mobility, handover, session, and routing inconsistencies accumulated over the preceding temporal context. The convergence of models near attack onset also shows why aggregate AUROC alone may underestimate the importance of genuinely early detection capability.

Table 10 evaluates whether the learned temporal representation preserves sufficient information to estimate not only the existence of future IRDA risk but also its temporal proximity. Conventional tabular methods exhibit the largest errors, with Random Forest producing an MAE of 1320 ms and RMSE of 1915 ms, whereas sequential and graph-based models progressively reduce these errors. The Temporal Graph Transformer provides the strongest baseline with an MAE of 492 ms, RMSE of 824 ms, and $R^{2}=0.969$. HATGT improves these values to 381 ms, 663 ms, and $R^{2}=0.980$, corresponding to approximately 22.6% lower MAE and 19.5% lower RMSE relative to the closest competitor. To distinguish temporal estimation accuracy from the influence of zero-valued targets, the table additionally presents positive-only regression metrics for samples satisfying $T_{i}>0$, representing 16.67% of the target distribution, while the remaining 83.33% have zero-valued targets. The tabulated positive-only MAE and RMSE for HATGT are 897 ms and 1364 ms, compared with 1121 ms and 1703 ms for the Temporal Graph Transformer, corresponding to reductions of approximately 20.0% and 19.9%, respectively. Random Forest exhibits substantially larger positive-only errors of 2957 ms MAE and 3943 ms RMSE. The improvement is also preserved across the error distribution: median absolute error decreases from 352 to 271 ms and P90 error decreases from 1290 to 1015 ms. These results indicate that handover-aware temporal modeling improves both average estimation accuracy and robustness against larger time-to-event prediction errors, although this benefit increases inference latency from 3.02 to 3.46 ms.

**Figure 8:**
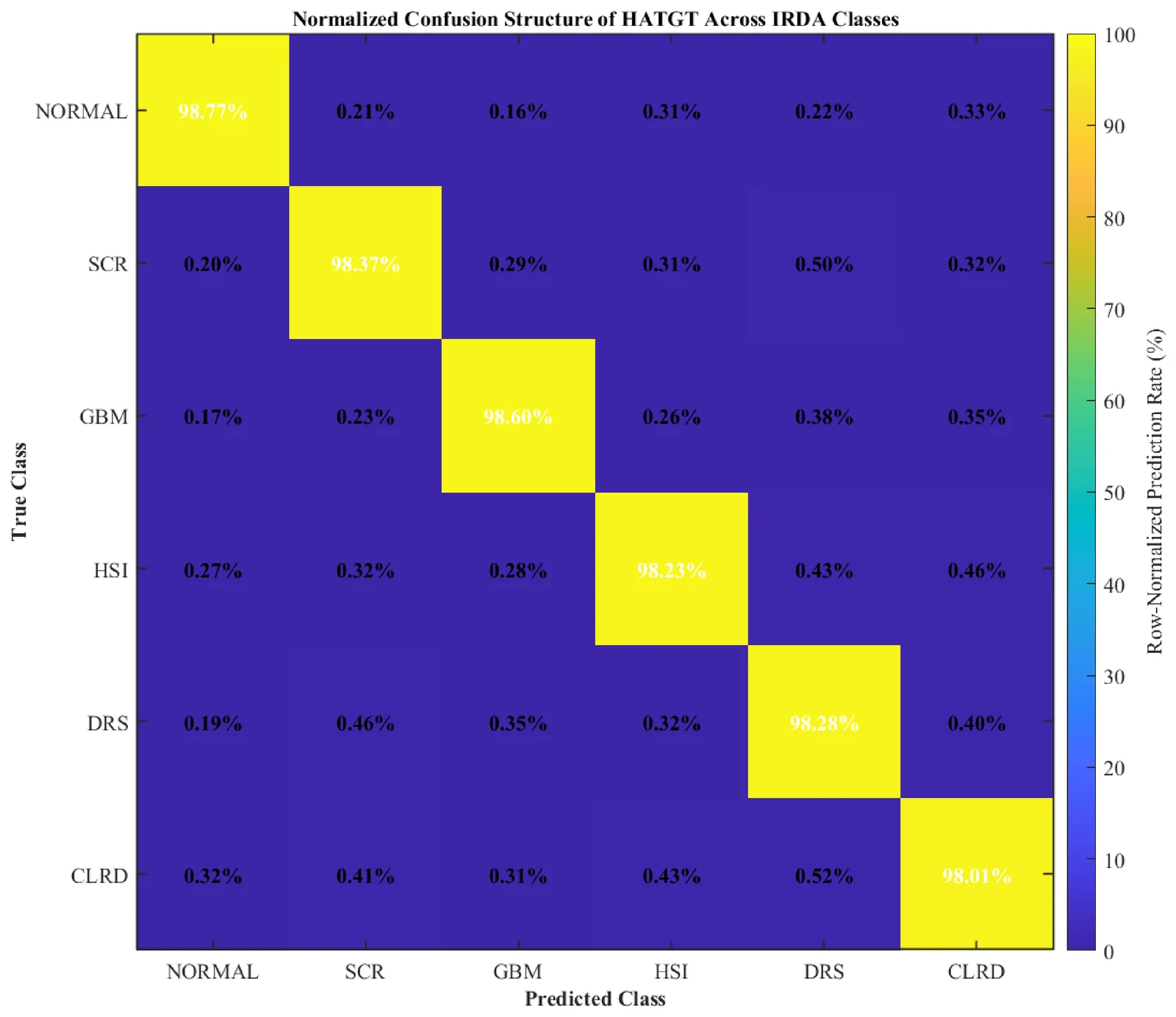
Row-normalized confusion structure of HATGT across NORMAL behavior and the five IRDA attack classes. Values indicate the percentage of each true class assigned to the corresponding predicted class.

A more detailed look at the classification errors that remain among the six behavioral categories is given in Figure 8, the normalized confusion structure. The diagonal stands out from the matrix, indicating that the coordinated low-rate desynchronization and NORMAL behavior had the highest correct classification rates of 98.01% and 98.77%, respectively, while there was strong separation between the legitimate mobility-induced transitions and adversarial identity–route inconsistencies. Notably, there is no off-diagonal confusion that exceeds about 0.52%. The biggest discrepancy that can be seen is between the coordinated low-rate desynchronization and delayed route synchronization, and this is because of the fact that they both have the property of slowly changing route-state inconsistencies and do not require any abrupt manipulations of the state. The same small residual confusion is observed for the delayed synchronization and coordinated low-rate behavior in handover-state injection, as these behaviors rely on the same type of transitional context. On the other hand, manipulation of the gateway is still quite localized, and achieves high recognition accuracy of 98.60%. The results show the ability of HATGT to preserve the temporal and relational structure of the attacks and to withstand legitimate transient inconsistencies caused by mobility, routing convergence and handover recovery.

**Figure 9:**
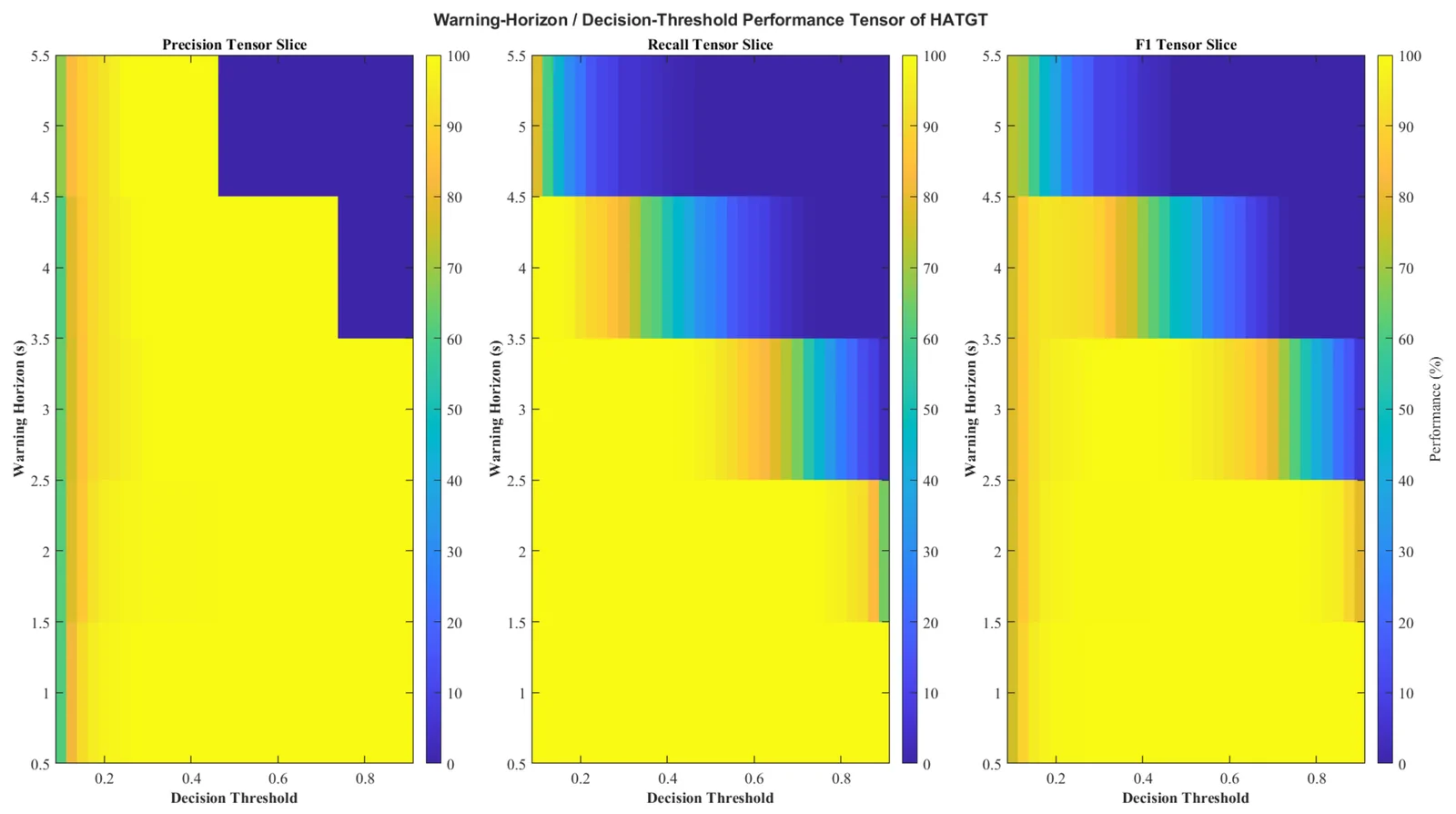
Warning-horizon/decision-threshold performance tensor of HATGT for five-second IRDA forecasting, showing precision, recall, and F1-score as joint functions of prediction horizon and decision threshold.

Figure 9 characterizes the operating sensitivity of the HATGT early-warning head by jointly varying the prediction horizon and classification threshold. A clear interaction is observed between these two parameters. At shorter warning horizons, particularly approximately 1–3 s before attack onset, broad regions of the threshold space preserve very high precision, recall, and F1, indicating that precursor evidence becomes increasingly discriminative as IRDA activation approaches. At longer horizons of approximately 4–5 s, performance becomes substantially more threshold-sensitive because early precursors are weaker and less distinguishable from legitimate handover or route-convergence fluctuations. Increasing the decision threshold improves precision over part of this region but progressively suppresses recall, producing the corresponding degradation in F1 at aggressive operating points. This tensor therefore demonstrates why a single fixed-threshold metric cannot fully characterize early-warning capability. Instead, HATGT provides a tunable security operating region in which the threshold can be selected according to the required compromise among early detection, false alarms, and missed future attacks.

Table 11 quantitatively demonstrates the differential contributions of HATGT’s architectural components across attack attribution, early-warning prediction, and time-to-IRDA estimation. Removing causal temporal attention produces the largest degradation among the individual architectural component removals, reducing multiclass Macro-F1 by 2.50 percentage points and early-warning F1 by 8.65 percentage points, while increasing regression MAE from 381 to 698 ms, corresponding to an 83.20% increase. Removing relation-aware graph encoding reduces Macro-F1 by 1.76 points and early-warning F1 by 5.54 points, while increasing regression MAE by 60.63%. Similarly, removing the handover-aware encoder decreases Macro-F1 by 1.43 points and early-warning F1 by 4.38 points, with a 50.39% increase in MAE. These results indicate that causal temporal attention is particularly important for modeling the progression toward IRDA onset, while relation-aware graph learning and explicit handover-state encoding provide complementary information for detecting inconsistencies among access, session, gateway, and forwarding states.

**Figure 10:**
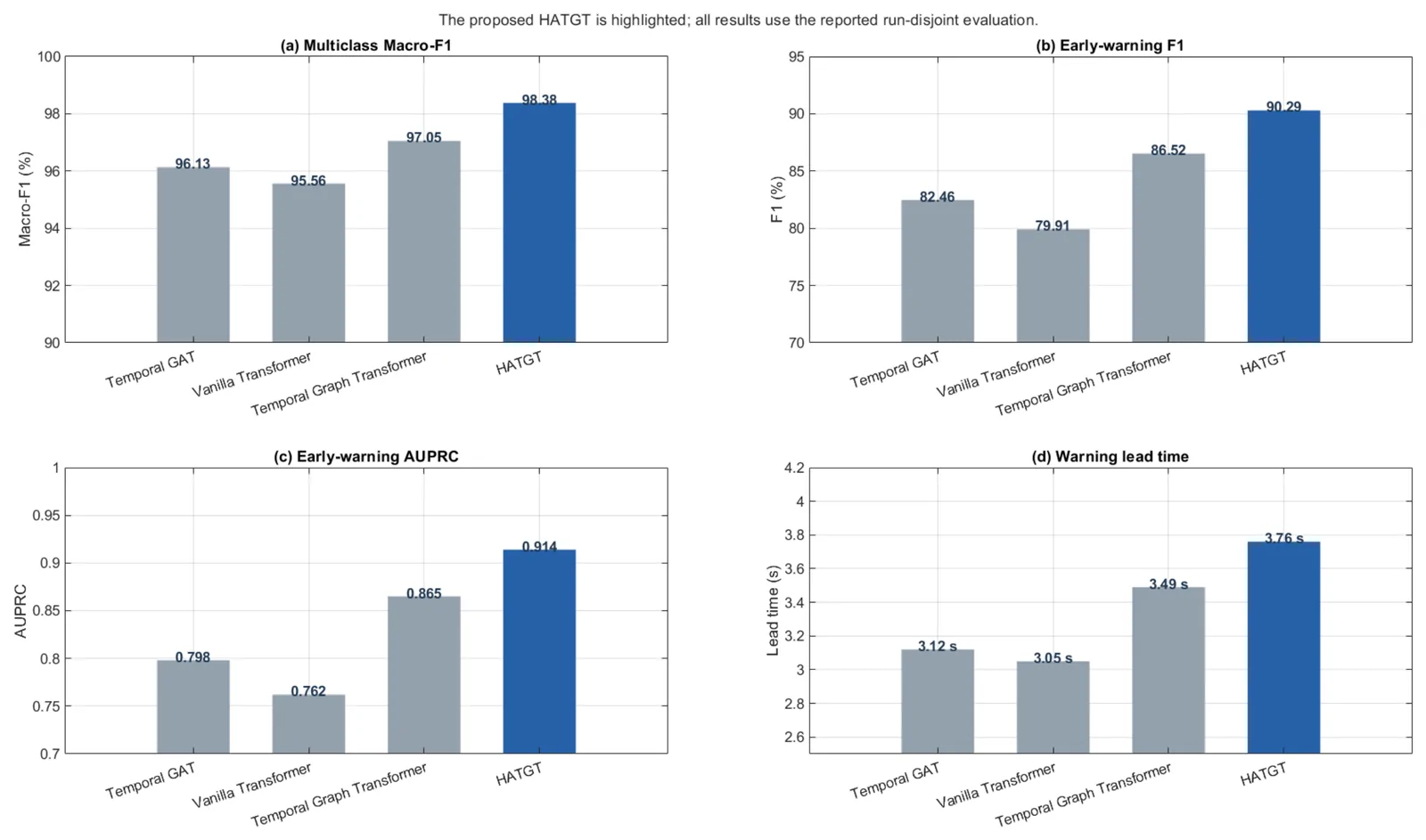
Focused comparison of HATGT against representative temporal baselines under the run-disjoint evaluation protocol, showing multiclass Macro-F1, early-warning F1, AUPRC, and mean warning lead time.

The graph-only and temporal-only variants exhibit greater performance degradation, with early-warning F1 reductions of 14.15 and 9.47 percentage points and relative MAE increases of 113.12% and 89.24%, respectively. Feature-group removal further demonstrates the sensitivity of the framework to cross-layer information: simultaneously excluding anchor, routing, handover, and session features reduces early-warning F1 by 17.66 percentage points and increases MAE by 145.67%. These findings show that the joint representation is more effective than the evaluated restricted variants under the investigated simulation configuration. Nevertheless, the ablation differences quantify conditional performance sensitivity rather than independent or additive causal contributions, and the regression results should be interpreted according to the target population used for MAE computation as shown in Figure 10.

A summary of the four coordinated security goals of HATGT is presented in Table 3 to illustrate the difference between the proposed multi-task architecture and the typical single output intrusion classifiers. For current-state binary detection, target_binary_irda combines the five IRDA mechanisms into 83,330 observations of malware (83.33%) and 16,670 observations of NORMAL (16.67%). The five IRDA categories consist of about the same number of observations, not the naturally occurring attack prevalence in operational TN–NTN situations, but a deliberately constructed benchmark for controlled attack-attribution analysis. These reported binary F1 scores, which range from  98–99%, are therefore not to be interpreted as the operational detection effectiveness, but rather as a measure of performance under the attack-enriched benchmark. Assuming the prevalence of IRDA to be 0.1%, and the conditional detection rate and false-positive rate to be constant, the expected precision is 5.22%, which means a false-discovery proportion of 94.78%. The prevalence adjusted estimates show that the positive predictive value and alert reliability varies considerably with the attack frequency and this variable is important under realistic operating conditions when reporting the precision, the false positive rate, the negative predictive value and the false alert rate (per 10,000 observations).

**Figure 11:**
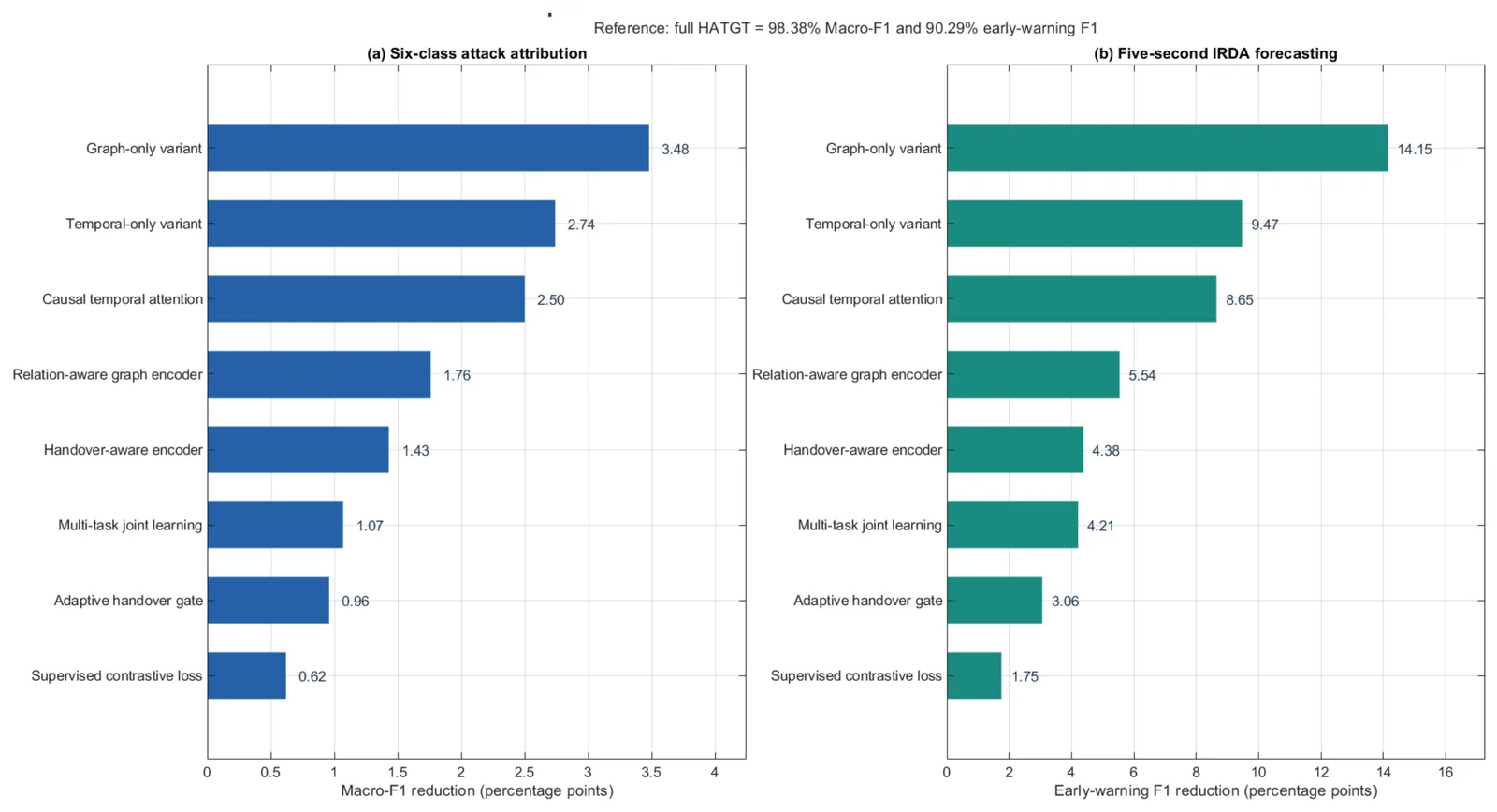
Performance degradation under architecture-level ablations relative to the complete HATGT framework: (**a**) multiclass Macro-F1 reduction and (**b**) five-second early-warning F1 reduction. Values are expressed as absolute percentage-point differences from the full model.

Figure 11 illustrates the performance degradation caused by architectural ablations relative to the complete Handover-Aware Temporal Graph Transformer (HATGT), which achieves 98.38% Macro-F1 for six-class attack attribution and 90.29% F1 for five-second IRDA forecasting. The graph-only variant produces the largest reductions, reaching 3.48 and 14.15 percentage points, respectively, followed by the temporal-only variant with reductions of 2.74 and 9.47 points. Among individual component removals, excluding causal temporal attention causes the greatest deterioration, decreasing Macro-F1 by 2.50 points and early-warning F1 by 8.65 points. Removing relation-aware graph encoding or the handover-aware encoder also reduces performance, highlighting the complementary value of relational and mobility-transition information. Multi-task joint learning, adaptive handover gating, and supervised contrastive loss provide additional improvements. Overall, forecasting shows greater sensitivity to architectural ablation than attack attribution, supporting the importance of jointly modeling temporal evolution, network relationships, and handover states under the investigated simulation conditions. These reductions represent conditional performance differences rather than independent, additive component contributions.

**Figure 12:**
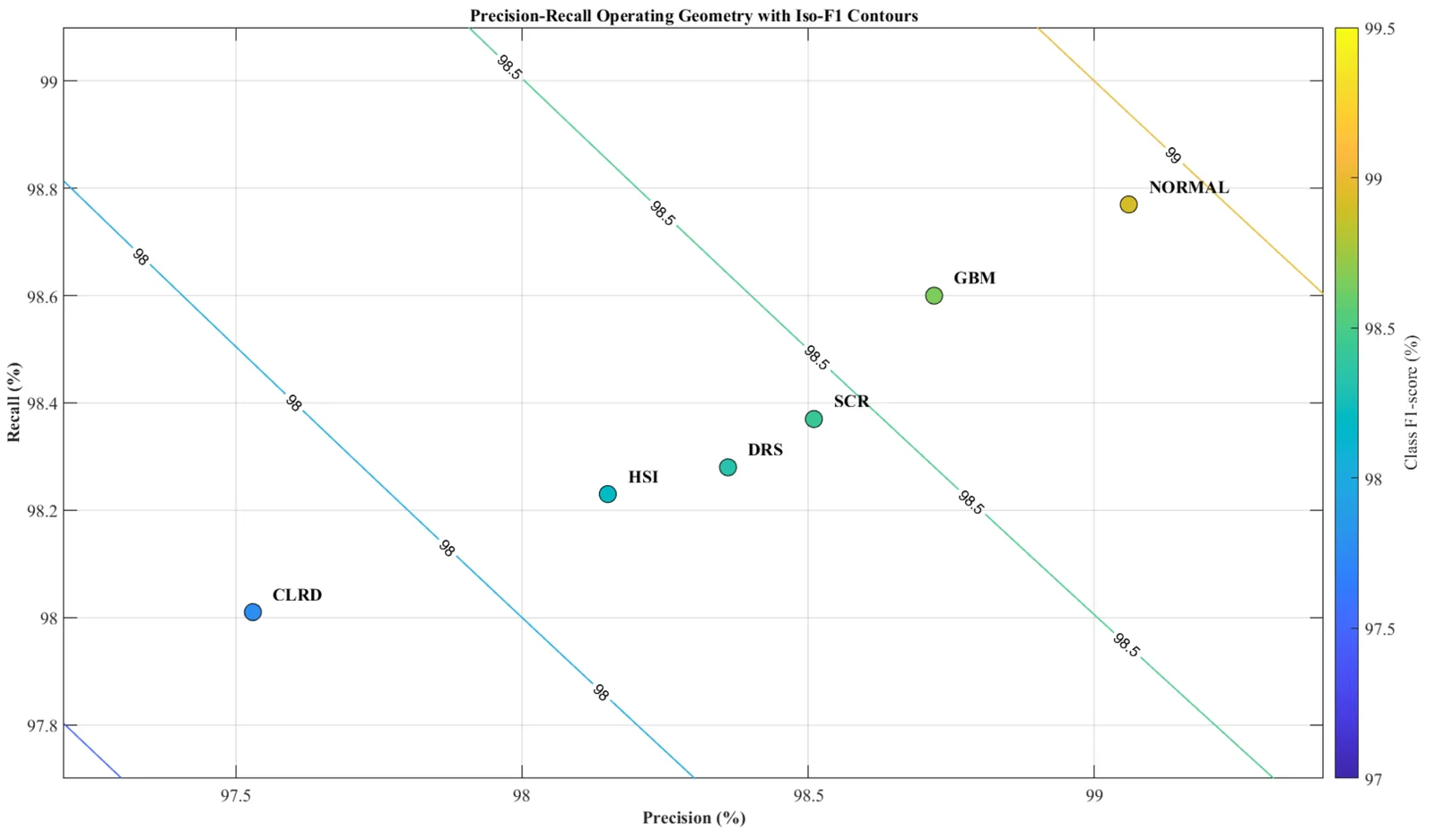
Precision–recall operating geometry of the proposed HATGT across the six behavioral classes, with iso-F1 contours illustrating equivalent harmonic precision–recall performance regions.

To examine the operating geometry of HATGT per class, each class is plotted in precision–recall space and iso-F1 contours are overlaid, as shown in Figure 12. The high detection completeness is illustrated by the compactness of the 6 classes in the upper right corner, while the low false positive contamination is illustrated by the sparse (small) number of “noise” classes in the lower left corner. While the precision and recall of NORMAL remain close to 99% F1 contour at 99.06% and 98.77% respectively, it exhibits the best operating point. NORMAL has the best operating point with precision and recall close to the 99% F1 contour at 99.06% and 98.77% respectively. The route-anchor manipulation shows a fairly unique relational signature with a precision of 98.72% and a recall of 98.60% when binding to a gateway. SCR, DRS and HSI are clustered together in the middle, and all of them have similar temporal features, but are well-differentiated on the basis of discrimination. Overall, CLRD is located the furthest from the upper right bound since its accuracy drops to 97.53% while its recall is still at 98.01%. It is important to note that the general geometry of low-rate attacks is that the problem is not so much that they are missed as that they are mis-detected, where false-positive ambiguity is a challenge.

**Figure 13:**
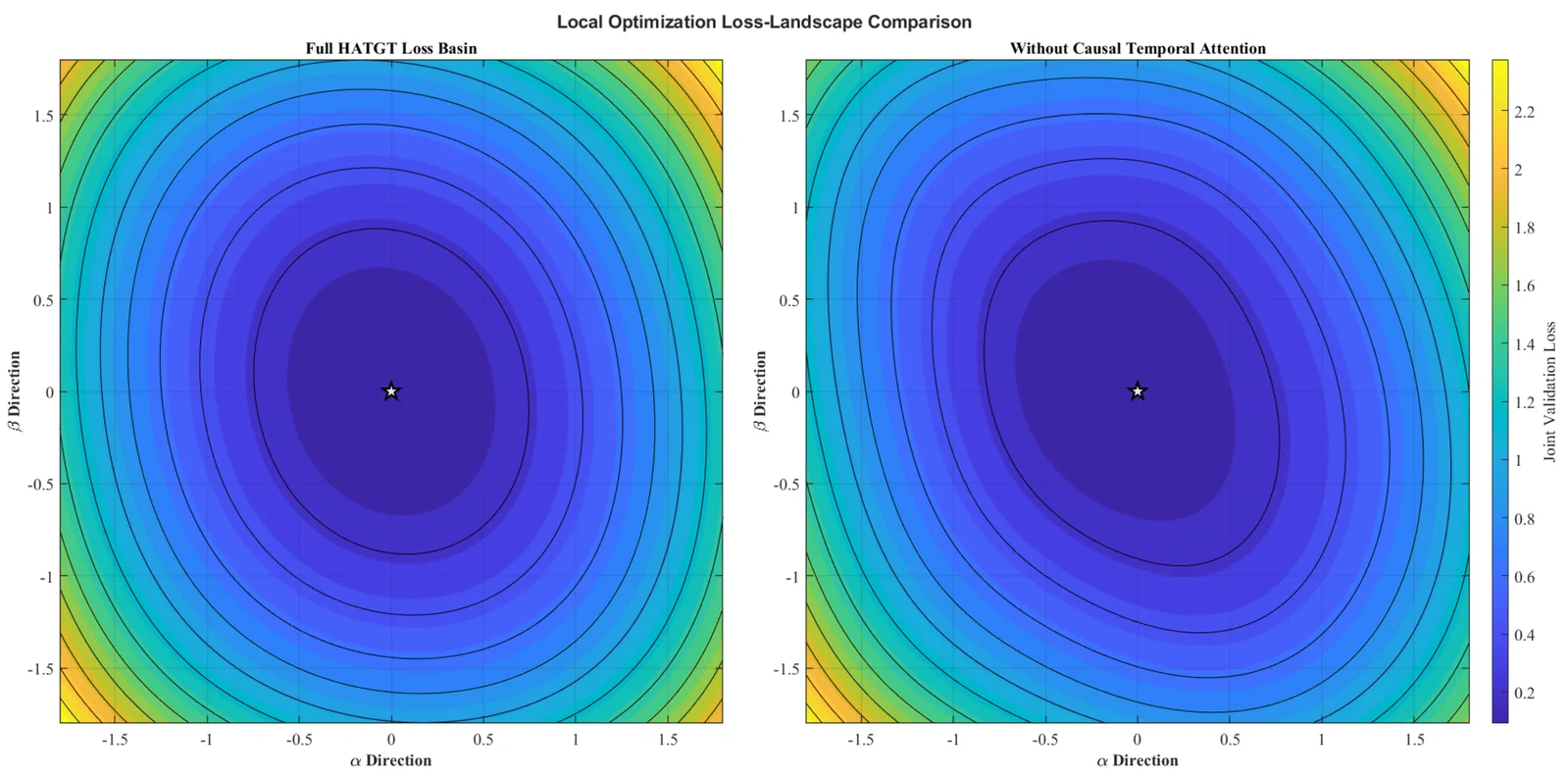
Local optimization loss-landscape comparison between the complete HATGT architecture and the variant without causal temporal attention. The α and β axes represent two local parameter-space perturbation directions around the optimized solution.

To gain further insight into the geometry of the optimization around the learned solution, we examine the local joint-loss surface of the full HATGT and the architecture obtained with the causal temporal attention removed, in Figure 13. The full model has a rather well-rounded, regularly shaped basin around this optimum, and fairly widely spaced contours of low loss with little directional distortion. Such geometry corresponds to lower sensitivity to small variations of the parameters and suggests a general behavior towards a relatively stable neighborhood within the parameter space. Causally removing temporal attention creates a more anisotropic landscape, with the low-loss area now becoming more elongated and shifted by direction. This behavior indicates higher coupling of the directions of the parameters and higher sensitivity to perturbations when there is no explicit temporal causality. This observation is of specific interest for IRDA detection, since the observation of route inconsistencies in temporal order helps identify whether the behavior is a natural transient handover or adversarial behavior/desynchronization. Therefore, causal temporal modeling also leads to a more structured optimization geometry that enables stable multi-task learning.

## 5. Conclusions and Future Work

This study presented a Handover-Aware Temporal Graph Transformer (HATGT) for detecting, classifying, forecasting, and estimating the timing of Identity–Route Desynchronization Attacks (IRDAs) in 6G-oriented LEO satellite–terrestrial networks. The framework was developed using ns-3.48 and 5G-LENA v5.1, incorporating heterogeneous UE mobility, terrestrial gNBs, LEO satellites, NTN gateways, routing evolution, session transitions, and four handover types. The term “6G-oriented” refers to anticipated architectural characteristics of future integrated TN–NTN systems rather than a finalized 6G air interface or an operational 6G deployment. Five IRDA mechanisms were modeled using 100,000 synchronized observations from 60 independent simulation runs with leakage-safe temporal windows and run-disjoint partitioning. HATGT integrates relation-aware graph encoding, handover-aware modeling, adaptive fusion, causal temporal attention, supervised contrastive learning, and multi-task prediction. Under the evaluated simulation conditions, HATGT achieved 98.42% multiclass accuracy, 98.38% Macro-F1, and 90.29% early-warning F1, with an average warning lead time of 3.76 s under the selected 5 s prediction horizon. However, this warning interval does not establish sufficient time for operational mitigation, and the reported 3.46 ms latency represents model inference on an NVIDIA RTX 4070 GPU rather than end-to-end detection latency. The ablation findings indicate that architectural mechanisms and informative cross-layer features jointly contribute to predictive performance, while the dominance of zero-valued regression targets requires careful interpretation of aggregate time-to-IRDA errors and separate positive-target evaluation. Importantly, the 42/9/9 run-disjoint protocol evaluates generalization across independent stochastic realizations of a common network configuration rather than across substantially different topologies, traffic distributions, mobility regimes, or attack-generation frameworks. Consequently, the findings provide simulation-level evidence of the potential applicability of HATGT to future satellite–terrestrial security systems without establishing operational deployment readiness or validated performance in actual 6G networks. Future work will investigate cross-topology and out-of-distribution generalization, reduced-feature and shortcut-learning evaluations, positive-target regression accuracy, alternative early-warning horizons, closed-loop mitigation effectiveness, and end-to-end detection and response latency. Additional studies will explore online adaptation, explainable temporal-graph reasoning, resource-constrained edge processing, adversarial robustness, hardware-in-the-loop platforms, and operational TN–NTN testbeds to assess transferability, scalability, and practical deployment feasibility.

## Figures and Tables

**Table 1:** Comparative synthesis of representative satellite, SAGIN, dynamic-graph, and mobility-aware security approaches and the research gap addressed by the proposed HATGT framework.

Study	Network/Security Domain	Core Method	Evaluation Source	Temporal/Graph Modeling	Handover Awareness	Identity–Route Synchronization	Primary Security Objective	Main Limitation Relative to HATGT
Azar et al. [17]	Satellite-network intrusion detection	Sequential Forward Selection with RF, ANN, LSTM, and GRU	STIN; UNSW-NB15	Sequential learning	No	No	Traffic-level attack classification	Does not jointly model mobility, handover, session, gateway, and forwarding-route consistency.
Driouch et al. [18]	Satellite CAN security	Distributed satellite/ground IDS with machine learning classifiers	Satellite CAN attack scenarios	Limited	No	No	Onboard CAN attack detection	Focuses on CAN-frame attacks rather than TN–NTN mobility-dependent cross-layer state synchronization.
Shi et al. [19]	Satellite-network intrusion detection	BiLSTM with stacked sparse autoencoder	STIN; UNSW-NB15	Sequential temporal learning	No	No	Traffic attack classification	Does not explicitly model heterogeneous UE, satellite, gateway, anchor, route, and handover relationships.
He et al. [20]	Satellite–terrestrial intrusion detection	Federated conditional diffusion, differential privacy, and intrusion classification	OMNeT++/INET STIN environment	Distributed learning	No	No	Intrusion detection under non-IID traffic	Addresses traffic-distribution heterogeneity rather than temporal inconsistency among handover, session, anchor, and routing states.
Xie et al. [21]	SAGIN intrusion detection	Smart-contract matching, semi-supervised voting, and distributed processing	Simulated SAGIN environment	Limited	No	No	Rapid traffic-oriented attack detection	Does not model mobility-dependent synchronization among identity/session, handover, gateway, and forwarding- route states.
Liu and Guo [22]	Dynamic network intrusion detection	Dynamic GNN, graph convolution, multi-head attention, and line-graph transformation	UNSW-NB15; NF-ToN-IoT-v2	Dynamic graph learning	No	No	Real-time intrusion detection	Uses generic communication graphs without explicit TN–NTN handover phases, control/data anchors, or identity–route synchronization.
Dewa et al. [23]	LEO satellite mobility management	Distributed graph message passing	MATLAB LEO simulation	Graph-based mobility modeling	Yes	No	Handover optimization	Optimizes legitimate handover decisions but does not identify malicious post-handover session–route inconsistencies.
Naz et al. [24]	LEO secure routing	MPNN-based trust learning with trust-constrained Dijkstra routing	OneWeb and Telesat simulations	Graph-based routing	No	Partial	Malicious-node detection and secure routing	Focuses on trusted route selection rather than temporal synchronization between legitimate UE identity/session and forwarding states.
Uddin et al. [25]	SAGIN-enabled IIoT security	Autoencoder, attention-based trust aggregation, time-varying GNN, and DQN	CSE-CIC-IDS2018	Time-varying graph reasoning	No	No	Cross-tier intrusion detection and mitigation	Does not explicitly represent handover-dependent synchronization among UE identity/session, gateway, and forwarding-route states.
Yao and Koirala [26]	Dynamic IIoT–5G security	Temporal GNN with contrastive learning, adversarial regularization, and fault-consistency optimization	Edge-IIoTset; ToN-IoT; IoT-23	Temporal graph learning	No	No	Fault-tolerant intrusion detection	Does not incorporate LEO/TN–NTN handover semantics, identity–route synchronization, or joint present and future IRDA prediction.
Proposed HATGT	6G-oriented LEO satellite–terrestrial security	Relation-aware heterogeneous graph encoding, handover-aware state encoding, causal temporal Transformer, and supervised contrastive learning	ns-3.48/5G-LENA v5.1 TN–NTN simulation	Heterogeneous temporal graph learning	Yes	Yes	Six-class IRDA attribution, binary detection, 5 s early warning, and time-to-IRDA estimation	Jointly models mobility, handover, session, anchor, gateway, and forwarding-route evolution to identify and forecast IRDA.

**Table 2:** Integrated technical configuration of the 6G-oriented TN–NTN environment, IRDA behavior space, and HATGT processing architecture.

System Layer	Component	Parameter	Standard/Mode	Configuration	Graph Entity/Relation	State Scope	HATGT Mapping
A. Simulation and dataset configuration							
Simulation	Platform	Simulator	Discrete-event	ns-3.48	System	All	Data generation
Simulation	NR framework	Implementation	NR/NTN	5G-LENA v5.1	System	All	Data generation
Simulation	Scenario	Network type	6G-oriented	TN–NTN	System	All	Topology generation
Simulation	Runs	Independent runs	Run-disjoint	60	Run	All	Partitioning
Simulation	Run partition	Train/Val/Test	Run-disjoint	42/9/9	Run	Common configuration	Within-configuration evaluation
Dataset	Candidate pool	Maximum UE-state records	Before curation	300,000	UE-state	All	Candidate observations
Dataset	Observations	Retained sample count	After filtering/balancing	100,000	UE-state	NORMAL/IRDA	Model input
Dataset	Columns	Feature space	Tabular + relational	50	UE-state	All	Feature construction
Dataset	Predictors	Network states	Leakage-safe	46	UE-state	All	Predictive input
Dataset	Targets	Learning outputs	Multi-task	4	UE-state	NORMAL/IRDA	Prediction heads
Dataset	Simulated UEs	Network population	Active topology	60	UE	All	Simulation context
Dataset	Monitored UEs	Retained identifiers	Learning population	50	UE	NORMAL/IRDA	Graph grouping
Dataset	Class balance	Six-class support	Class-aware sampling	16,670/5×16,666	UE-state	NORMAL/IRDA-1–5	Multiclass target
Dataset	Sampling	Resolution	Temporal	0.5 s	UE trajectory	All	Sequence builder
Dataset	Timeline	Retained observation interval	Local timestamp	0–49.5 s	UE trajectory	All	Temporal ordering
B. Terrestrial NR access configuration							
TN-RAN	gNB	Node count	NR	3	GNB	All	Graph node
TN-RAN	Carrier	Frequency	NR	∼3.5 GHz	UE–GNB	All	Radio encoder
TN-RAN	Channel	Bandwidth	NR	∼20 MHz	UE–GNB	All	Radio encoder
TN-RAN	Waveform config.	Numerology	NR	μ=1	UE–GNB	All	Radio context
TN-RAN	Duplexing	Frame mode	TDD	TDD	UE–GNB	All	Radio context
TN-RAN	gNB	Tx power	NR	∼43 dBm	GNB	All	Radio context
C. LEO non-terrestrial configuration							
NTN	Satellite	Node count	LEO	6	SAT	All	Graph node
NTN	Orbit	Altitude	LEO	∼550 km	SAT	All	NTN geometry
NTN	Carrier	Frequency	NR-NTN	∼700 MHz	UE–SAT	All	Radio encoder
NTN	Channel	Bandwidth	NR-NTN	∼5 MHz	UE–SAT	All	Radio encoder
NTN	Gateway	Node count	NTN	2	GW	NORMAL/IRDA	Graph node
NTN	Link	Propagation delay	Time-varying	0.318–6.251 ms	UE–SAT	All	$x_{u,t}^{radio}$
NTN	Link	Doppler	LEO dynamics	−19–19 kHz	UE–SAT	All	$x_{u,t}^{radio}$
NTN	Geometry	Elevation	LEO dynamics	0–85°	UE–SAT	All	$x_{u,t}^{radio}$
NTN	Geometry	Serving distance	Dynamic	0.08–1700 km	UE–SAT/GNB	All	$x_{u,t}^{radio}$
D. Core, gateway, and edge configuration							
Core	UPF	Node count	User plane	1	UPF	All	Graph node
Edge	Application	Server count	Edge service	2	EDGE	All	Graph node
Control plane	Anchor	Control binding	Dynamic	UPF-01/GW-01/GW-02	UE–Anchor	NORMAL/IRDA	Relation encoder
Data plane	Anchor	Data binding	Dynamic	UPF-01/GW-01/GW-02	UE–Anchor	NORMAL/IRDA	Relation encoder
E. UE mobility and handover configuration							
Mobility	UE population	Simulated UEs	Heterogeneous	60	UE	All	Mobility context
Mobility	Static	UE share	Mobility profile	∼12%	UE	NORMAL context	$x_{u,t}^{mob}$
Mobility	Pedestrian	UE share	Mobility profile	∼48%	UE	NORMAL context	$x_{u,t}^{mob}$
Mobility	Pedestrian	Speed	Mobility profile	0.8–2.0 m/s	UE	NORMAL context	$x_{u,t}^{mob}$
Mobility	Vehicular	UE share	Mobility profile	∼40%	UE	NORMAL context	$x_{u,t}^{mob}$
Mobility	Vehicular	Speed	Mobility profile	7–27 m/s	UE	NORMAL context	$x_{u,t}^{mob}$
Handover	Trigger	Hysteresis	A3	∼3 dB	UE–Access	NORMAL/IRDA-sensitive	$f_{\mathrm{HO}}$
Handover	Trigger	Nominal TTT	A3	∼80 ms	UE–Access	NORMAL/IRDA-sensitive	$f_{\mathrm{HO}}$
Handover	Trigger	Dataset TTT	A3	80/100/128/ 160 ms	UE–Access	NORMAL/IRDA-sensitive	$T_{u}^{TTT}$
Handover	A3 state	Margin	Continuous	−21.15–2.97 dB	UE–Access	NORMAL/IRDA-sensitive	$a_{u,t}^{A3}$
Handover	TTT state	Progress	Temporal	0–175 ms	UE–Access	NORMAL/IRDA-sensitive	$p_{u,t}^{TTT}$
Handover	Phase	State	Mobility	STABLE	UE	NORMAL baseline	$e_{\mathrm{phase}}$
Handover	Phase	State	Mobility	PREPARATION	UE	NORMAL/precursor	$e_{\mathrm{phase}}$
Handover	Phase	State	Mobility	EXECUTION	UE	NORMAL/IRDA-sensitive	$e_{\mathrm{phase}}$
Handover	Phase	State	Mobility	RECOVERY	UE	NORMAL/IRDA-sensitive	$e_{\mathrm{phase}}$
Handover	Transition	Access pair	Mobility	TN–TN	GNB→GNB	NORMAL/IRDA	$e_{\mathrm{type}}$
Handover	Transition	Access pair	Mobility	TN–NTN	GNB→SAT	NORMAL/IRDA	$e_{\mathrm{type}}$
Handover	Transition	Access pair	Mobility	NTN–TN	SAT→GNB	NORMAL/IRDA	$e_{\mathrm{type}}$
Handover	Transition	Access pair	Mobility	NTN–NTN	SAT→SAT	NORMAL/IRDA	$e_{\mathrm{type}}$
Handover	History	Time since last HO	Elapsed state duration	0–79.88 s	UE	NORMAL/IRDA-sensitive	$\tau_{u,t}^{lastHO}$
Handover	History	Last-HO duration	Temporal	85–220 ms	UE	NORMAL/IRDA-sensitive	$d_{u,t}^{lastHO}$
Handover	History	HO count	Cumulative	0–11	UE	NORMAL/IRDA-sensitive	$c_{u,t}^{HO}$
F. Transport, traffic, and QoS configuration							
Transport	Protocol	Transport type	TCP/UDP	TCP, UDP	UE–EDGE	All	Traffic context
Application	Traffic	Profile	CONTROL	CONTROL	UE–EDGE	NORMAL/IRDA	Context encoder
Application	Traffic	Profile	TELEMETRY	TELEMETRY	UE–EDGE	NORMAL/IRDA	Context encoder
Application	Traffic	Profile	VIDEO	VIDEO	UE–EDGE	NORMAL/IRDA	Context encoder
Application	Traffic	Profile	BACKGROUND	BACKGROUND	UE–EDGE	NORMAL/IRDA	Context encoder
Traffic	Load	Packet rate	Dynamic	7.02–425.45 pps	Flow	All	$x_{u,t}^{qos}$
Traffic	Throughput	Data rate	Dynamic	0.0119–3.9738 Mbps	Flow	All	$x_{u,t}^{qos}$
QoS	Latency	RTT	End-to-end	8.21–68.81 ms	UE–EDGE	All	$x_{u,t}^{qos}$
QoS	Queueing	Queue delay	Dynamic	0.30–15.99 ms	Path	All	$x_{u,t}^{qos}$
QoS	Delay variation	Jitter	Dynamic	0.54–12.58 ms	Path	All	$x_{u,t}^{qos}$
QoS	Reliability	Packet loss	Ratio	0.0016–0.0150	Flow	All	$x_{u,t}^{qos}$
QoS	Transport	Retransmission	Ratio	0.00127–0.01069	Flow	All	$x_{u,t}^{qos}$
Traffic	Concurrency	Active flows	Count	1–6	UE	All	$x_{u,t}^{qos}$
G. Routing and session-state configuration							
Routing	Route state	Version	Dynamic	1–33	Route	NORMAL/IRDA	$x_{u,t}^{route}$
Routing	Route state	Route age	Accumulated state age	50–153,472 ms	Route	NORMAL/IRDA-sensitive	$r_{u,t}^{age}$
Routing	Path state	Changes/10 s	Temporal	0–12	Path	NORMAL/IRDA-sensitive	$x_{u,t}^{route}$
Session	State	Session phase	Dynamic	ACTIVE	UE session	NORMAL/IRDA	$e_{\mathrm{session}}$
Session	State	Session phase	Dynamic	PREPARING	UE session	NORMAL/precursor	$e_{\mathrm{session}}$
Session	State	Session phase	Dynamic	MIGRATING	UE session	NORMAL/IRDA-sensitive	$e_{\mathrm{session}}$
Session	State	Session phase	Dynamic	RECOVERING	UE session	NORMAL/IRDA-sensitive	$e_{\mathrm{session}}$
Session	Context	Session age	Accumulated state age	20.81–469.55 s	UE session	NORMAL/IRDA-sensitive	$x_{u,t}^{session}$
H. IRDA security-state configuration							
Security	Baseline	Class	Legitimate	NORMAL	Temporal graph	NORMAL	Multiclass target
Security	IRDA-1	Mechanism	Context replay	STALE CONTEXT	Session/Route	IRDA	Multiclass target
Security	IRDA-2	Mechanism	Binding manipulation	GATEWAY BINDING	Anchor relation	IRDA	Multiclass target
Security	IRDA-3	Mechanism	State injection	HANDOVER STATE	Handover relation	IRDA	Multiclass target
Security	IRDA-4	Mechanism	Delayed synchronization	ROUTE SYNC	Route/Anchor	IRDA	Multiclass target
Security	IRDA-5	Mechanism	Low-rate desync.	COORDINATED	Multi-state	IRDA	Multiclass target
Security	Detection	Binary state	Current-state	NORMAL/IRDA	UE temporal state	Binary	${\hat{y}}_{bin}$
Security	Forecast	Prediction horizon	Early warning	5 s	Pre-attack state	Future IRDA	${\hat{y}}_{future}$
Security	Regression	Time-to-event	Temporal risk	0–59996 ms	Pre-attack state	Future IRDA	$\hat{T}$
I. Heterogeneous graph representation							
Graph	Node set	Entity types	Heterogeneous	UE/GNB/SAT/ GW/UPF/EDGE	$V_{t}$	All	Node embedding
Graph	Serving	Relation	Typed	SERVED_BY	UE→GNB/SAT	NORMAL/IRDA	Relation attention
Graph	Candidate	Relation	Typed	CANDIDATE_ACCESS	UE→GNB/SAT	NORMAL/precursor	Relation attention
Graph	Satellite relay	Relation	Typed	RELAYED_BY	SAT→GW	NORMAL/IRDA	Relation attention
Graph	Forwarding	Relation	Typed	FORWARDED_TO	GW→UPF	NORMAL/IRDA	Relation attention
Graph	Service	Relation	Typed	CONNECTED_TO	UPF→EDGE	All	Relation attention
Graph	Handover	Relation	Typed	HANDOVER_TO	UE→GNB/SAT	NORMAL/IRDA	Relation attention
J. HATGT learning architecture							
HATGT	Input	Feature groups	Concatenation	Radio/Mob./HO/ Route/QoS/Session	$x_{u}^{t}$	All	Input state
HATGT	Spatial layer	Graph encoder	Relation-aware	$f_{\mathrm{HGE}}$	$G_{t}$	All	Spatial encoding
HATGT	Mobility layer	HO encoder	Handover-aware	$f_{\mathrm{HO}}$	$q_{u,t}^{HO}$	All	Mobility encoding
HATGT	Fusion layer	Adaptive gate	Sigmoid	$\gamma_{u,t}$	Graph + HO	All	State fusion
HATGT	Temporal layer	Attention	Causal MHA	Mask *M*	$G_{t-L+1:t}$	All	$f_{\mathrm{CT}}$
HATGT	Temporal layer	Position	Learnable	$p_{k}$	Sequence state	All	Temporal embedding
HATGT	Temporal layer	Processing	Transformer	Residual + LN + FFN	Sequence state	All	Temporal encoder
HATGT	Representation	Regularization	Sup. contrastive	$L_{con}$	Latent state	NORMAL + 5 IRDA	Class separation
HATGT	Head 1	Classification	Softmax	6 classes	$h_{u,t}^{*}$	NORMAL + 5 IRDA	${\hat{y}}_{cls}$
HATGT	Head 2	Detection	Sigmoid	Binary	$h_{u,t}^{*}$	NORMAL/IRDA	${\hat{y}}_{bin}$
HATGT	Head 3	Forecasting	Sigmoid	5 s horizon	$h_{u,t}^{*}$	Future IRDA	${\hat{y}}_{future}$
HATGT	Head 4	Regression	Softplus	Time-to-IRDA	$h_{u,t}^{*}$	Pre-attack	$\hat{T}$
HATGT	Loss	Classification	Cross-entropy	$L_{cls}$	Output	6 classes	Joint loss
HATGT	Loss	Detection	Binary	$L_{bin}$	Output	NORMAL/IRDA	Joint loss
HATGT	Loss	Forecasting	Class-weighted	$L_{pred}$	Output	Future IRDA	Joint loss
HATGT	Loss	Regression	Smooth-L1	$L_{reg}$	Output	Time-to-IRDA	Joint loss
HATGT	Optimization	Objective	Multi-task	$L_{total}$	Θ	All	Joint training
HATGT	Optimization	Optimizer	AdamW	Learning rate η	Θ	All	Parameter update
HATGT	Validation	Criterion	Run-disjoint	Macro-F1	$D_{val}$	All	Model selection
HATGT	Testing	Protocol	Run-disjoint	Unseen runs	$D_{te}$	All	Final evaluation

**Table 3:** Multi-task security targets, temporal semantics, class distributions, and HATGT output-head configurations for the 100,000-record TN–NTN IRDA dataset.

Target	Security Objective	Label Semantics	Horizon/ Domain	Output Space	Support	Distribution	HATGT Head	Optimization/Evaluation
target_binary_ irda	Current-state detection	State at *t*	Instantaneous	{0,1} 0: NORMAL 1: IRDA	NORMAL: 16,670 IRDA: 83,330	16.67% 83.33% ≈ 1:5	Sigmoid ${\hat{y}}_{bin}$	$L_{bin}$ Accuracy, Precision, Recall, F1, AUROC, AUPRC, FPR, FNR Benchmark prevalence: 83.33% IRDA; binary F1 approximately 98–99% (benchmark only). At 0.1% assumed prevalence, projected precision: 5.22%. Base-rate sensitivity: Precision, Recall, F1, AUPRC, FPR, NPV, and false alarms per 10,000 observations
target_future_ irda_5s	Early-warning prediction	Future IRDA onset Relative to a distinct attack-onset event	(t,t+5s]	{0,1} 0: No onset 1: Onset	0: 98,282 1: 1718	98.282% 1.718% ≈ 57.21:1	Sigmoid ${\hat{y}}_{future}$	Class-weighted $L_{pred}$ Precision, Recall, F1, AUROC, AUPRC, FAR, Lead Time Event-level Precision, Event-level Recall, Event Lead Time, Missed Events, Duplicate Alerts/Event
target_time_to_ irda_ms	Time-to-event estimation	Next IRDA onset interval	Temporal regression	$T_{u,t}\geq 0$ 0–59,996 ms Positive: 500–59,996 ms	Zero: 83,330 Positive: 16,670	83.33% zero 16.67% positive	Softplus ${\hat{T}}_{u,t}$	Smooth-L1 $L_{reg}$ log(1+T) training transform MAE, RMSE, $R^{2}$
target_attack_ type	Attack attribution	State/mechanism at *t*	Six-class	NORMAL IRDA-1–IRDA-5	NORMAL: 16,670 IRDA-1–5: 5×16,666	16.670% 5×16.666% Near-balanced	Softmax ${\hat{y}}_{cls}$	Cross-entropy $L_{cls}$ Macro-P, Macro-R, Macro-F1, Weighted-F1, AUROC, AUPRC

**Table 4:** Reproducibility matrix of the HATGT data pipeline, architecture, optimization, loss configuration, and computational evaluation, including observation-level and event-level early-warning evaluation protocols.

Block	Processing Unit	Input/Span	Latent Size	Layers/Steps	Heads	Dropout	Control Parameter	Execution Setting
A. Data, graph, and temporal-sequence formation								
Observation	UE state	0.5 s/sample	46 predictors	100 timestamps	1 stream	0.00	100,000 retained	60 independent runs
Graph snapshot	UE-centered graph	1 timestamp	192	6 node types	4 relation heads	0.15	6 relation types	Dynamic reconstruction
Temporal history	Same UE/run	L=20 snapshots	192	20 steps	6 temporal heads	0.15	stride = 1	10 s context
Forecast target	Future IRDA state	5 s horizon	1 probability	10 future steps	1 output	0.00	weight = 57.21	Causal prediction
Partition	Run-level split	42/9/9 runs	70/15/15%	3 subsets	1 split	0.00	seed = 2026	Run-disjoint
B. HATGT spatial, mobility, and temporal architecture								
Entity embedding	Categorical states	Per category	32	1 embedding	1 stream	0.10	learnable	Shared embedding table
Graph encoder	$f_{\mathrm{HGE}}$	1 graph snapshot	192	2 layers	4 heads	0.15	48 dim/head	Relation-aware attention
HO encoder	$f_{\mathrm{HO}}$	$q_{u,t}^{HO}$	128→192	2 layers	1 stream	0.15	GELU activation	Mobility-state projection
Adaptive fusion	$\gamma_{u,t}$	Graph + HO	192	1 gate	1 gate vector	0.00	sigmoid	Element-wise fusion
Temporal encoder	$f_{\mathrm{CT}}$	20 fused states	192	3 layers	6 heads	0.15	32 dim/head	Causal MHA
Temporal FFN	Transformer FFN	Per temporal layer	384	3 blocks	1 FFN/block	0.15	GELU + LN	Residual connection
Contrastive map	$g(h^{*})$	Per temporal sample	128	2 layers	1 projection	0.10	τ=0.07	$\ell_{2}$ normalization
Multiclass head	${\hat{y}}_{cls}$	$h_{u,t}^{*}$	6 outputs	1 layer	1 head	0.10	Softmax	NORMAL + 5 IRDA
Binary head	${\hat{y}}_{bin}$	$h_{u,t}^{*}$	1 output	1 layer	1 head	0.10	Sigmoid	Current IRDA state
Forecast head	${\hat{y}}_{future}$	$h_{u,t}^{*}$	1 output	1 layer	1 head	0.10	Sigmoid	5 s early warning
Regression head	$\hat{T}$	$h_{u,t}^{*}$	1 output	1 layer	1 head	0.10	Softplus	Time-to-IRDA
C. Joint optimization and regularization								
Optimizer	All Θ	Batch = 64	2.84 M params	120 epochs	1 optimizer	0.15	$\eta =2\times 10^{-4}$	AdamW
Weight decay	Trainable weights	All layers	2.84 M params	120 epochs	1 schedule	0.15	$10^{-4}$	Decoupled regularization
Gradient control	Global gradients	Every update	All parameters	1 clip/update	1 norm	0.00	clip = 1.0	$\ell_{2}$ norm clipping
Early stopping	$D_{\mathrm{val}}$	9 validation runs	Macro-F1	12 epochs	1 criterion	0.00	patience = 12	Best checkpoint
Classification loss	$L_{cls}$	6 classes	6 logits	1 objective	1 head	0.00	$\lambda_{cls}=1.00$	Cross-entropy
Detection loss	$L_{bin}$	NORMAL/IRDA	1 logit	1 objective	1 head	0.00	$\lambda_{bin}=0.50$	Binary CE
Forecast loss	$L_{pred}$	5 s target	1 logit	1 objective	1 head	0.00	$\lambda_{pred}=1.50$	Weighted BCE
Regression loss	$L_{reg}$	log(1+T)	1 value	1 objective	1 head	0.00	$\lambda_{reg}=0.50$	Smooth-L1
Contrastive loss	$L_{con}$	128-D projection	128	1 objective	1 projection	0.10	$\lambda_{con}=0.10$	Supervised contrastive
D. Reproducibility and runtime protocol								
Simulation seeds	ns-3 runs	60 realizations	60 seeds	1 seed/run	1 stream	0.00	900,001–900,060	Reproducible replay
Learning seed	Model/split	All experiments	1 seed	1 initialization	1 stream	0.00	2026	Deterministic mode
Model footprint	Full HATGT	1 model	2.84 M	3 temporal layers	6 heads	0.15	214 MB	Inference memory
Warm-up	Forward pass	1 UE sequence	L=20	100 passes	6 heads	0.00	batch = 1	Timing excluded
Timed inference	Forward pass	1 UE sequence	L=20	1000 passes	6 heads	0.00	batch = 1	3.46 ms/sample
Timing statistic	Mean latency	1000 trials	1 sample/trial	1000 measures	1 model	0.00	device sync.	I/O excluded
Training host	Ubuntu 22.04 LTS	Intel Core i7-13700K	NVIDIA RTX 4070	12 GB VRAM	32 GB RAM	CUDA 12.1	PyTorch 2.4.1	Python 3.11.9
E. Observation-level and event-level early-warning evaluation								
Observation unit	UE-time prediction	0.5 s sampling	1 probability	5 s horizon	1 score/step	Disabled	1718 positive labels	Timestamp-level scoring
Observation metrics	Binary forecasts	Held-out timestamps	0/1 target	Per observation	1 prediction	Disabled	P/R/F1, AUROC, AUPRC	Threshold-based FAR
Attack event	IRDA onset	Non-IRDA to IRDA	1 event ID	1 onset/episode	1 UE/run	Disabled	Run, UE, onset time	Repeated active states excluded
Warning grouping	Alert aggregation	Pre-onset interval	1 warning episode	0–5 s before onset	First valid alert	Disabled	Same UE and onset	Duplicate alerts merged
Event precision	Warning episodes	Matched/ unmatched	Event TP/FP	1 credit/event	1 event score	Disabled	$TP_{e}/\left(TP_{e}+FP_{e}\right)$	Requires event counts
Event recall	Actual IRDA onsets	Detected/ missed	Event TP/FN	1 onset/event	1 event score	Disabled	$TP_{e}/\left(TP_{e}+FN_{e}\right)$	Requires event counts
Event F1	Aggregated alerts	Event P and R	1 F1 value	Event aggregation	1 event score	Disabled	$2P_{e}R_{e}/\left(P_{e}+R_{e}\right)$	Requires event counts
Warning lead time	First valid warning	Before IRDA onset	Time in seconds	Detected events	1 lead/event	Disabled	$t_{e}^{on}-t_{e}^{first}$	3.76 s reported mean
False warnings	Unmatched episodes	No onset in 5 s	Event FP count	After merging	1 episode	Disabled	Outside valid onset match	Separate from timestamp FAR

**Table 5:** Overall six-class IRDA classification performance under the run-disjoint test protocol.

Model	Accuracy (%)	Macro-P (%)	Macro-R (%)	Macro-F1 (%)	Weighted-F1 (%)	AUROC	AUPRC
Random Forest	90.84	90.72	90.63	90.61	90.83	0.972	0.926
XGBoost	92.73	92.64	92.51	92.50	92.72	0.981	0.944
BiLSTM	94.36	94.21	94.13	94.12	94.34	0.986	0.958
GRU	93.98	93.85	93.76	93.74	93.96	0.985	0.955
GAT	95.11	94.99	94.91	94.90	95.09	0.989	0.965
Temporal GAT	96.28	96.17	96.12	96.13	96.27	0.992	0.975
Vanilla Transformer	95.72	95.61	95.55	95.56	95.71	0.991	0.970
Temporal Graph Transformer	97.16	97.09	97.04	97.05	97.15	0.995	0.983
Proposed HATGT	98.42	98.39	98.38	98.38	98.42	0.998	0.992

**Table 6:** Per-class classification performance of the proposed HATGT for normal behavior and individual IRDA mechanisms.

Class	Precision (%)	Recall (%)	F1-Score (%)	AUROC	AUPRC	Dataset Support
NORMAL	99.06	98.77	98.91	0.999	0.995	16,670
IRDA Stale Context Replay	98.51	98.37	98.44	0.998	0.992	16,666
IRDA Gateway Binding Manipulation	98.72	98.60	98.66	0.999	0.995	16,666
IRDA Handover-State Injection	98.15	98.23	98.19	0.997	0.988	16,666
IRDA Delayed Route Synchronization	98.36	98.28	98.32	0.998	0.990	16,666
IRDA Coordinated Low-Rate Desynchronization	97.53	98.01	97.77	0.995	0.985	16,666
Macro Average	98.39	98.38	98.38	0.998	0.991	100,000
Weighted Average	98.39	98.38	98.38	0.998	0.991	100,000

**Table 7:** Binary current-state IRDA detection performance under the run-disjoint benchmark.

Model	Accuracy (%)	Precision (%)	Recall (%)	F1-Score (%)	AUROC	AUPRC	FPR (%)	FNR (%)
Random Forest	94.18	97.02	95.89	96.45	0.971	0.991	14.36	4.11
XGBoost	95.62	97.63	97.10	97.36	0.981	0.994	11.80	2.90
BiLSTM	96.78	98.14	98.00	98.07	0.987	0.996	9.30	2.00
GRU	96.39	97.95	97.70	97.82	0.985	0.995	10.20	2.30
GAT	97.17	98.26	98.35	98.30	0.990	0.997	8.70	1.65
Temporal GAT	98.01	98.87	98.74	98.80	0.994	0.998	5.65	1.26
Vanilla Transformer	97.62	98.62	98.52	98.57	0.992	0.997	6.88	1.48
Temporal Graph Transformer	98.55	99.33	98.93	99.13	0.997	0.999	3.35	1.07
Proposed HATGT	98.95	99.64	99.10	99.37	0.999	0.999	1.80	0.90

**Table 8:** HATGT sensitivity analysis under low-prevalence IRDA conditions.

IRDA Prevalence (%)	Precision (%)	Recall (%)	F1-Score (%)	FPR (%)	NPV (%)	False Alarms/10,000
0.1	5.22	99.10	9.92	1.80	99.999	179.82
0.5	21.67	99.10	35.56	1.80	99.995	179.10
1.0	35.74	99.10	52.53	1.80	99.991	178.20
2.0	52.91	99.10	68.99	1.80	99.981	176.40
5.0	74.34	99.10	84.95	1.80	99.952	171.00
10.0	85.95	99.10	92.06	1.80	99.898	162.00

**Table 9:** Five-second early-warning IRDA prediction performance under the run-disjoint test protocol.

Model	Precision (%)	Recall (%)	F1-Score (%)	AUROC	AUPRC	False Alarm (%)	Lead Time (s)	Miss Rate (%)
Random Forest	61.40	57.80	59.55	0.902	0.516	0.64	2.11	42.20
XGBoost	67.90	63.70	65.73	0.924	0.584	0.53	2.32	36.30
BiLSTM	75.80	72.40	74.06	0.953	0.681	0.40	2.86	27.60
GRU	73.60	70.80	72.17	0.947	0.659	0.44	2.71	29.20
GAT	78.40	74.10	76.19	0.961	0.716	0.36	2.54	25.90
Temporal GAT	84.30	80.70	82.46	0.976	0.798	0.27	3.12	19.30
Vanilla Transformer	81.70	78.20	79.91	0.969	0.762	0.31	3.05	21.80
Temporal Graph Transformer	88.10	85.00	86.52	0.986	0.865	0.20	3.49	15.00
Proposed HATGT	91.40	89.20	90.29	0.992	0.914	0.15	3.76	10.80

**Table 10:** Time-to-IRDA estimation performance of the evaluated models.

			Positive-Only	Positive-Only				
Model	MAE (ms)	RMSE (ms)	MAE (ms)	RMSE (ms)	$R^{2}$	Median AE (ms)	P90 AE (ms)	Inference Time (ms)
Random Forest	1320	1915	2957	3943	0.846	980	3120	0.42
XGBoost	1085	1602	2451	3308	0.892	790	2640	0.58
BiLSTM	824	1289	1813	2627	0.926	602	2110	1.41
GRU	876	1348	1934	2751	0.919	640	2210	1.17
GAT	746	1185	1687	2412	0.938	530	1920	1.76
Temporal GAT	598	981	1349	2018	0.957	421	1590	2.43
Vanilla Transformer	672	1068	1484	2157	0.948	468	1730	2.09
Temporal Graph Transformer	492	824	1121	1703	0.969	352	1290	3.02
Proposed HATGT	381	663	897	1364	0.980	271	1015	3.46

**Table 11:** Architecture- and feature-level ablation analysis of HATGT, including absolute classification and forecasting performance reductions, relative regression-error increases, computational efficiency, and sensitivity to feature-group removal.

Configuration	Macro-F1 (%)	ΔMacro-F1 (pp)	Binary F1 (%)	Early F1 (%)	ΔEarly F1 (pp)	Early AUPRC	MAE (ms)	ΔMAE (%)	Parameters (M)	Latency (ms)	Memory (MB)
**Full HATGT**	98.38	0.00	99.37	90.29	0.00	0.914	381	0.00	2.84	3.46	214
w/o Relation-Aware Graph Encoder	96.62	1.76	98.12	84.75	5.54	0.842	612	60.63	2.12	2.57	182
w/o Handover-Aware Encoder	96.95	1.43	98.39	85.91	4.38	0.858	573	50.39	2.56	3.17	201
w/o Adaptive Handover Gate	97.42	0.96	98.74	87.23	3.06	0.879	521	36.75	2.74	3.30	208
w/o Causal Temporal Attention	95.88	2.50	97.96	81.64	8.65	0.798	698	83.20	2.02	2.38	176
w/o Contrastive Loss	97.76	0.62	99.02	88.54	1.75	0.893	455	19.42	2.68	3.36	210
w/o Multi-Task Joint Learning	97.31	1.07	98.61	86.08	4.21	0.861	534	40.16	2.45	3.08	198
Graph-Only Variant	94.90	3.48	97.30	76.14	14.15	0.731	812	113.12	1.46	1.86	156
Temporal-Only Variant	95.64	2.74	97.72	80.82	9.47	0.784	721	89.24	1.71	2.11	164
**Targeted Feature-Group Ablation and Sensitivity Analysis**											
w/o Anchor-State Features	97.21	1.17	98.48	86.83	3.46	0.871	548	43.83	2.79	3.39	210
w/o Routing-State Features	96.74	1.64	98.17	84.92	5.37	0.846	617	61.94	2.78	3.37	209
w/o Handover-State Features	97.03	1.35	98.33	85.76	4.53	0.857	581	52.49	2.76	3.34	207
w/o Session-State Features	97.46	0.92	98.69	87.41	2.88	0.882	503	32.02	2.80	3.40	211
w/o Anchor + Routing Features	95.93	2.45	97.61	81.78	8.51	0.801	704	84.78	2.72	3.25	202
w/o Anchor + Routing + HO + Session Features	92.84	5.54	95.26	72.63	17.66	0.681	936	145.67	2.61	3.01	193
Identity–Route Consistency Rule	–	–	91.72	–	–	–	–	–	–	0.08	–

## Data Availability

The TN–NTN IRDA dataset used in this study was generated specifically for this research using ns-3.48 and the 5G-LENA v5.1. The generated dataset, simulation configurations, and supporting experimental data are available from the corresponding authors upon reasonable request.
